# Lymphocyte to monocyte ratio predicts survival and is epigenetically linked to miR-222-3p and miR-26b-5p in diffuse large B cell lymphoma

**DOI:** 10.1038/s41598-023-31700-x

**Published:** 2023-03-25

**Authors:** Ayman Mohamed Metwally, Ameera Abdel Hamed Mahmoud Kasem, Magda Ismail Youssif, Safia Mohammed Hassan, Abdel Hady A. Abdel Wahab, Lobna Ahmed Refaat

**Affiliations:** 1 Technology of Medical Laboratory Department, College of Applied Health Science Technology, Misr University for Science and Technology, 77, Almotamayez District, 6th October, Egypt; 2 Department of Histochemistry and Cell Biology, Medical Research Institute, Alexandria University, Alexandria, Egypt; 3 Department of Cancer Biology, National Cancer Institute, Cairo University, Cairo, Egypt; 4 Clinical Pathology Department, National Cancer Institute, Cairo University, Cairo, Egypt

**Keywords:** Cancer, Genetics, Molecular biology, Biomarkers

## Abstract

Diffuse large B-cell lymphoma (DLBCL) is the most common type of non-Hodgkin lymphoma. 10–20% of the patients present with bone marrow (BM) involvement which predicts a worse survival. This study aimed to determine the prognostic significance of serum miR-222-3p, miR-26b-5p, EBV-miR-BHRF1-2-5p, and EBV-miR-BHRF1-2-3p and correlate their levels to clinical and haematological markers in DLBCL with special emphasis on the lymphocyte-monocyte ratio (LMR) and neutrophil-monocyte ratio. We also studied the role of BM BMI1 and PIM2 proteins in predicting BM infiltration. Serum miRNAs were studied on 40 DLBCL and 18 normal individuals using qRT-PCR. BMI1 and PIM2 proteins were studied on BM biopsies by immunohistochemistry. The results were correlated with clinical and follow-up data. All the studied miRNAs were dysregulated in DLBCL serum samples. BMI1 and PIM2 were expressed in 67% and 77.5% of BM samples, respectively. LMR was significantly associated with disease-free survival (DFS) (*P* = 0.022), miR-222-3P (*P* = 0.043), and miR-26b-5p (*P* = 0.043). EBV-miR-BHRF1-2-3p was significantly correlated to haemoglobin level (*P* = 0.027). MiR-222-3p, miR-26b-5p, and EBV-miR-BHRF1-2-5p expressions were significantly correlated to each other (*P* = 0.001). There was no significant correlation between the studied markers and follow-up data. LMR is a simple method for predicting survival in DLBCL. MiR-222-3p and miR-26b-5p may be implicated in an immunological mechanism affecting patients’ immunity and accordingly influence LMR. The correlation between miR-222-3p, miR-26b-5p, and EBV-miR-BHRF1-2-5p may indicate a common mechanism among the 3 miRNAs that may explain DLBCL pathogenesis.

## Introduction

B-cell lymphomas are a heterogeneous group of lymphoproliferative malignancies originating from B cells with an unknown pathogenesis^1^. The classification of B cell lymphomas depends on the recognition of characteristic genetic anomalies that deregulate the expression of oncogenes or tumour suppressor genes^2^. B-cell non-Hodgkin lymphoma (NHL) is derived from mature B-cells and accounts for approximately 70–90% of lymphoid neoplasms worldwide and 4% of all new cancers each year^3^.

DLBCL is the most common lymphoid malignancy, accounting for 30–40% of adult lymphomas^4^. Based on their cell of origin, there are 2 distinct subtypes of DLBCL that differ in gene expression profiles: the germinal centre B cell-like (GCB) and the activated B cell-like (ABC) DLBCL^5^.

Patients are diagnosed depending on a group of clinical, laboratory, radiologic, and pathologic assessments^6^. DLBCL is diagnosed based on the histopathological examination of an excisional or incisional biopsy of a peripheral lymph node. If the lymph node is not accessible, core needle biopsy (CNB) and fine needle aspiration (FNA), in conjunction with IHC, FC, or cytogenetic analyses, may be sufficient for diagnosis^7^.

Despite advancements in DLBCL treatment, about 40% of patients relapse after treatment or are refractory to treatment^8^. A better understanding of the biology of DLBCL and more assessment of favourable or poor prognoses are clearly needed to facilitate better treatment decisions in the future and improve patient outcomes^9^.

MicroRNAs (miRNAs) are small, single-stranded RNAs that are approximately 22 nucleotides in length. They down-regulate the expression of genes encoding proteins or long non-coding RNAs (lnc-RNAs) by inhibiting mRNA translation or by promoting target RNA degradation. It was found that more than 30% of human protein-coding genes are regulated by miRNAs^10^.

Many studies have indicated the role of miRNA in the process of carcinogenesis, as dysregulated miRNA expression results in a disturbance in gene expression, which in turn leads to an imbalance in normal regulatory pathways resulting in malignant transformation. Recent studies have identified the relationship between dysregulated miRNA and DLBCL^11^, and others have indicated an important role for circulating miRNAs in the diagnosis of DLBCL^12^.

MiRNA-222-3P is a microRNA that is deregulated in cancer^13^. Studies on DLBCL indicated an important role of miR-222 in DLBCL pathogenesis^14^. Although an abnormal serum level of miR-222-3p was recently detected in many types of cancer, such as thyroid tumours^15^, its serum level in DLBCL patients has not been studied.

Recently, miR-26b-5p was found to be downregulated in several types of solid tumors, including bladder cancer^16^, thyroid cancer^17^, and hepatocellular carcinoma^18^. Studies of miR-26b-5p on lymphoid malignancies are limited, one study on Burkitt lymphoma indicated downregulation of miR-26b-5p^19^, and another study showed high expression of miR-26b in Nodal marginal zone lymphoma (NMZL)^20^.

Epstein-Barr virus (EBV) miRNAs were first identified by cloning small RNAs from a Burkitt's lymphoma cell line infected with EBV, and 4% of the cloned small RNAs originated from EBV^21^. Three EBVs, BHRF1-1, -2, and -3, are derived from the BHRF1 cluster^22^. EBV-miR-BHRF1-1 is found in the promoter region, while EBV-miR-BHRF1-2 and EBV-miR-BHRF1-3 are found in the 3′ untranslated region (UTR) of the BHRF1 cluster^23^. EBV-mir-BHRF1-2 includes two types: EBV-mir-BHRF1-2-5p and EBV-mir-BHRF1-2-3p. Functional assays demonstrated that EBV miR-BHRF1-2-5p contributes to the growth of latently infected B cells through growth factor receptor-bound protein 2 (GRB2) regulations. Further, EBV miR-BHRF1-2-5p activities directly regulate virus reactivation triggered by BCR engagement^24^.

Although the oncovirus (EBV) is an etiological factor in lymphoma, the associated microRNAs EBV-miR-BHRF1-2-5p and EBV-miR-BHRF1-2-3p have not been well studied in DLBCL. Accordingly, we tested the expression of these 2 microRNAs in serum samples of DLBCL patients and evaluated the significance of these miRNAs in recurrence and response to therapy.

B lymphoma Mo-MLV insertion region 1 homolog (BMI1), a polycomb group (PcG) protein, acts as an epigenetic regulator and plays an essential role in the regulation of various cellular processes. Such as proliferation, differentiation, cancer stem cells, and self-renewal. It acts as an oncogene to promote carcinogenesis in cancers such as prostate, lung, ovarian, urinary bladder, lymphoma, mesothelioma, medulloblastoma, glioma, acute myeloid leukemia, and breast cancer^25^. In aggressive B cell lymphomas, BMI1 is expressed in dividing neoplastic cells, where its aberrant expression contributes to malignant transformation in DLBCL, BL, and MCL^26^.

The prodigal insertion site in Maloney murine leukaemia virus (PIM) proteins is a family of proto-oncogenes that contains three members, PIM1, PIM2, and PIM3, constituting a group of closely related short-lived serine/threonine kinases^27^. In DLBCL, increased PIM2 expression was associated with an aggressive clinical course, and accordingly, targeting PIM2 kinase is a rational approach in DLBCL treatment^28^.

Many studies have demonstrated the importance of LMR in predicting tumour recurrence^29^. The factors or mechanisms that regulate this ratio in cancer are not known. For this reason, we studied the 4 miRNAs as possible epigenetic influencers on both LMR and NMR. Especially because previous studies indicated an immune regulatory mechanism for these miRNAs, for example, miR-222 is linked to pathways essential for immune regulation^30–32^. The overexpression of miR-26b-5p is involved in HLA class I-mediated immune escape through reducing the expression of HLA class I cell surface antigens, inducing decreased T cell recognition in melanoma^33^, and triggering T cell responses in HCC^34^. However, EBV-related miRNAs directly suppress host antiviral immunity by interfering with antigen presentation and immune cell activation^35^.

Recent studies aim at studying non-invasive or minimally invasive techniques that can accurately diagnose and predict the survival of cancer patients. Among these techniques is the use of liquid biopsy, which allows the detection of nucleic acids released in the bloodstream and other biological fluids^36^. Liquid biopsy could be used for diagnosis and detecting recurrence in cancer patients^37^.

Actually, the rationale behind correlating the miRNA and protein markers as well as the haematological criteria of the patients, especially LMR, LMR, PLR, NMR, and SII, with the follow-up data of the patients is to find a simple, cheap, and minimally invasive method that predicts patients' response to therapy, recurrence, and survival.

The present study aims to detect the prognostic significance of serum miR-222-3p, miR-26b-5p, EBV-miR-BHRF1-2-5p, and EBV-miR-BHRF1-2-3p and the significance of BMI1 and PIM2 proteins to differentiate between infiltrative and non-infiltrative BM in DLBCL. And to correlate the expression levels of the 4 miRNAs to LMR and NMR in blood samples of the patients.

## Materials and methods

### Study design

Forty newly diagnosed diffuse large B-cell lymphoma (DLBCL) patients were involved in the study. At the time of sample retrieval, the patients had not received any type of therapy or had any type of infection. Serum and bone marrow samples were collected from each patient. Eighteen serum samples were collected from age- and sex-matched normal individuals. Micro RNA expression was studied in serum samples while the BMI1 and PIM2 protein expression was detected in the bone marrow samples. Clinicopathological data were collected, and patients were followed up for up to 2 years. Laboratory results were correlated to the clinicopathological criteria and follow-up results. The work has been carried out in accordance with the Code of Ethics of the World Medical Association (Declaration of Helsinki) for experiments involving humans. Approval was granted by the Ethics Committee of the National Cancer Institute, Cairo University (IRP approval no. 202101-2p-03001).

### Material

After acceptance by the ethical committee of the National Cancer Institute, Cairo University (IRP approval no. 202101-2p-03001), 40 archived serum samples and bone marrow biopsies were collected from DLBCL patients. The selected samples were collected from diagnosed patients before they received any type of therapy (during the time of sample collection, patients did not receive any type of treatment). The diagnosis was confirmed for all patients by the histopathological examination of the removed lymph nodes by two independent pathologists. Discrepancies and divergences of opinion were resolved through a third reviewer, a pathologist. Eighteen serum samples were collected from age- and sex-matched healthy individuals. Before enrolling in the study, the donors were asked if they had any blood disorders or a medical history of blood disorders. They were also asked for any signs that identify a bacterial or viral infection. In addition, blood samples were tested for the presence of bacterial and viral infections or any haematological problems. Paraffin blocks of bone marrow biopsies were sectioned at a thickness of 4 µm and were placed onto positively charged slides. 5 ml blood was collected from each patient and normal individual in plain gel vacationer tube, centrifuged and serum was collected and aliquoted in sterile, DNAase, RNAse free cryotubes then stored at − 80 °C.

All patient data were retrospectively collected from the patients’ files and included the following: Age, sex, histopathological data, hematological data (complete blood count (CBC), Lymphocyte Monocyte ratio (LMR) (LMR = absolute lymphocyte count/absolute monocyte count), Neutrophil Monocyte ratio (NMR) (NMR = absolute neutrophil count/absolute monocyte count), Platelet Lymphocyte ratio (PLR) (PLR = absolute platelet count/ absolute lymphocyte count), Neutrophil Lymphocyte ratio (NLR) (NLR = absolute neutrophil count/absolute lymphocyte count), Systemic immune-inflammation index (SII) (SII = platelet count × neutrophil count)/lymphocyte count), bone marrow samples, bone marrow biopsies, Immunophenotyping (CD20, CD3, CD30, and CD15), Biochemical examinations including lactate dehydrogenase (LDH) and beta 2 microglobulin (B2M) and serological data including Hepatitis C virus (HCV) (HCV Ab, HbsAg) and Human immunodeficiency virus (HIV) (HIV Ab). Follow-up data, including metastasis, response to therapy, recurrence, tumour progression, mortality, overall survival (OS), disease-free survival (DFS), and progression-free survival (PFS), were collected.

Regarding the treatment, patients received the R-CHOP chemotherapy regimen, consisting of cyclophosphamide 750 mg/m2, doxorubicin 50 mg/m2, vincristine 1.4 mg/m2, prednisone 100 mg/day, and rituximab 375 mg/m2.

### Methods

#### RNA extraction

Serum samples were thawed, and 200 l of each sample were subjected to total RNA extraction, including miRNA extraction, using the miRNeasy mini kit from Qiagen (catalogue no. 217004). The purity and concentration of the RNA were determined using Nanodrop technology from Thermo Scientific.

#### cDNA synthesis

cDNA was synthesized from RNA using miRCURY LNA RT Kit, Qiagen (cat. no. 339340). Briefly, 2–4 µl Template RNA (correspond to RNA isolated from 200 μl serum and eluted in 50 μl) was added to 2 μl 5 × miRCURY RT Reaction Buffer, 1 μl 10 × miRCURY RT Enzyme Mix, 0.5 μl UniSp6 RNA spike-in and completed to 10 μl RNase-free water. The reaction was Incubated at 42ºC for 60 min followed by Incubation at 95 °C for 5 min and then immediately kept at 4 °C.

#### Quantitative RT-PCR

cDNA was diluted by adding 290 μl RNase-free water to the 10 μl RT reaction. The following primers from Qiagen (cat. no. 339306) were used: YP00204551 for miR-222-3p, YP00204172 for miR-26b-5p, YP00205766 for EBV-miR-BHRF1-2-5p, YP00205854 for EBV-miR-BHRF1-2-3p and YP00203901 for SNORD38B (hsa). For each miRNA the following cocktail was used: 5 μl 2 × miRCURY SYBR Green Master Mix (cat. no. 339345), 0.05 μl ROX Reference Dye, 1 μl PCR primer mix, 3 μl diluted cDNA template, and RNase-free water. The 10 μl reaction mixture was dispensed into PCR plate wells and the plate was centrifuged to remove air bubbles. PCR reaction was done on Applied Biosystems ViiA 7 and the machine was programmed as follows:

**Table:** 

Step	Time	Temperature	Ramp rate
1-PCR initial heat activation	2 min	95 °C	Maximal/fast mode
2- Cycling:			
Denaturation	10 s	95 °C	Maximal/fast mode
Combined annealing/extension	60 s	56 °C	Maximal/fast mode
Number of cycles: 40			
3-Melting curve analysis		60–95 °C	

Samples were studied in duplicate. After ending the PCR program, CT (threshold cycles) values were collected and the fold change for each miRNA was calculated using 2^−ΔΔCT^ method^38^.

#### Immunohistochemical analysis for BMI1 and PIM2

BMI1 and PIM2 proteins were studied on the paraffin-embedded sections from bone marrow biopsies. Monoclonal antibodies for BMI1 from R&D Systems (UK) (Cat. MAB33342) and PIM2 from Santa Cruz Biotechnology (USA) (Cat. sc-13514) were added to the tissue section, and biotinylated goat anti-polyvalent was added as a secondary antibody. 3,3′-Diaminobenzidine (DAB) substrate was added and visualized by DAB chromogen UltraVision Quanto Detection System Anti-Polyvalent, HRP/DAB Thermo Scientific (Cat. no. TP-015-HD). For each sample, three slides were stained, and the mean score was detected.

##### Immunohistochemical Scoring

Slides were evaluated by two independent pathologists. Discrepancies between individuals were resolved through a third reviewer, a pathologist. In addition, the results were confirmed by re-evaluating the slides through Aperio imagingScope (Leica Biosystems Imaging) version 12.2.0.5092.

BMI1 was scored based on the method of Hayry et al.^39^. The scoring was based on the frequency of cells with positive nuclear staining (range, 0%–100%) and was divided into three categories: mild ( +), moderate (+ +), and high (+ + +), where immunostainings were grouped according to the percentage of positive tumour cell nuclei in the sample as follows: 0 = no staining, +  = mild (less than 20% of positive nuclei), +  +  = moderate (20–70% of positive nuclei), and +  +  +  = high (more than 70% of positive nuclei).

PIM2 expression was scored according to the method of Ren et al.^40^ and also classified as mild ( +), moderate (+ +), and high (+ + +). The percentages of positivity and intensity of staining were graded as follows: 0 = no staining (0–5%), +  = mild (6–25%), +  +  = moderate (26–75%), and +  +  +  = high (76–100%).

#### In-silico analysis for miR-222-3p, miR-26b-5p and ebv-miR-BHRF1-2-5p target prediction and pathways enrichment

MiRNAs target gene prediction was studied using DIANA-TarBase v8 database (Database for experimentally supported interactions)^41^. Overlapping and unique target genes were identified using Venn diagram (https://bioinformatics.psb.ugent.be/webtools/Venn/). Functional enrichment analysis of overlapping target genes was performed using ‘Metascape (https://metascape.org)^42^.

### Statistical data

Statistical analysis was done using IBM Statistical Package for Social Sciences (SPSS) version 22 (IBM Corp., Armonk, NY). Numerical data were expressed as median and range. Qualitative data were expressed as frequency and percentage. Pearson’s Chi-square test or Fisher’s exact test was used to examine the relation between qualitative variables. Quantitative data were tested for normality using the Kolmogorov–Smirnov test and the Shapiro–Wilk test. Data were found to be not normally distributed. Comparison between two groups was done using the Mann–Whitney test (non-parametric t-test). Comparison between 3 groups was done using Kruskal–Wallis test (non-parametric ANOVA). The Spearman-Rho method was used to test the correlation between numerical variables. Survival analysis was done using the Kaplan–Meier method, and comparison between two survival curves was done using the log-rank test. All tests were two-tailed. A *p*-value of 0.05 was considered significant.

### Ethical approval

This study was performed in line with the principles of the Declaration of Helsinki. Approval was granted by the Ethics Committee of the National Cancer Institute, Cairo University (IRP Approval No. 202101-2p-03001). Written informed consent was obtained from all subjects or their legal guardian(s).

## Results

The current study was done on 40 histopathologically proven DLBCL patients during the period from 2018 to 2020. Archived Serum and bone marrow samples were taken from the patients before treatment. Eighteen serum samples were collected from 18 age- and sex-matched healthy individuals. Follow-up data for up to 2 years, including disease-free and progression-free status as well as overall survival, were collected.

### Characteristics of the studied patients

Nineteen patients were male (47%), and 21 were female (53%). The median age of the patients was 51.5 (ranging from 20.0 to 80.0; 22). Seven patients had stage I or II (17.7%), 14 patients had stage III (35%), 16 patients had stage IV (40%), and three patients were missing (7.5%). Thirty-two patients had splenomegaly (80%), seven patients had a normal spleen (17.5%), and one patient was missing (2.5%). Fourteen cases (35%) had bone marrow infiltration, and 26 cases (65%) were negative. Twelve cases were positive for reticulin (30%) (4 stage I, 6 stage II, 1 stage III, 1 stage IV), 15 cases were nil (37.5%), and 13 cases were missing (32.5%). Seventeen cases were positive for B symptoms (42.5%), 22 were negative (55%) and 1 case was missing (2.5%). The median LDH was 405.5 (U/L) (range 171.0–2099). The median B2M was 3.06 mcg/mL (range 2.34–8.80). Thirty-five percent of the studied cases were positive for HCV-A (14 cases). Only one case was positive (2.5%) for HBsAg (Supplementary Table S1).

The median white blood cell count was 7.06 109/L (2.3–28.57), the median red blood cell count (RBCs) was 4.495 million cells/mcL (min. 2.98–max. 5.89), and the median haemoglobin (Hb) level was 11.45 g/dL (7.5–14.9). The median hematocrit level was 34.95% (22.5%–46.5%), the median platelet (PLT) count was 241.5 × 103/mm3 (40–757), the median neutrophils count was 59.35 × 109/L (4–90.9), the median lymphocytes count was 27.55 × 109/L (1–95), and the median monocytes count was 8.25 × 109/L (1–18.6). The median lymphocyte monocyte ratio (LMR) was 3 (0.1–95), the median neutrophil monocyte ratio (NMR) was 7.4 × 109/L (2.7–59), the median platelet lymphocyte ratio (PLR) was 134.274 (6.742–851.875), the median neutrophil lymphocyte ratio (NLR) was 2.131 (0.042–113.652), and the median systemic immune-inflammation index (SII) was 477.189 (7.705–9658.125).

### Follow-up data of the studied patients

Out of 40 cases, 35 received chemotherapy (87.5%). Seventeen cases were responsive to chemotherapy (48.6%), 12 cases were resistant (34.3%), 4 cases had a partial response (11.4%), and 2 cases did not complete their treatment (5.7%). Among the 17 responsive cases, 5 had recurrence (29.4%), and the remaining 12 were non-recurrent (70.6%). Seven out of 12 resistant cases had disease progression (58.3%), and the remaining five had non-progressive disease (41.7%). Twenty-nine patients were alive (72.5%), seven patients died (17.5%), and four were missing (10%). The median follow-up was 12.1 months, the median disease-free survival was 8 months (2–22 months), the median progression-free survival was 8 months (1.1–20.27 months), and the median overall survival was 15 months (0.06–24.4 months) (Supplementary Table S2).

### miRNAs were dysregulated in serum and the protein markers were expressed in the bone marrow of DLBCL patients

All the samples tested for miR-222-3p were dysregulated (100%), where 29 samples (72.5%) were up-regulated and 11 samples (27.5%) were down-regulated. Regarding miR-26b-5p, 29 samples were up (72.5%) and 11 were down (27.5%) regulated, for an overall dysregulation rate of 100%. EBV-miR-BHRF1-2-5p expression was dysregulated in 31 samples (77.5%), where 25 samples were up (62.5%), 6 were down (15%), and 9 samples were not differentially expressed (22.5%). For EBV-miR-BHRF1-2-3p, all the tested samples were dysregulated (100%); 27 samples were up-regulated (67.5%) and 13 (32.5%) were down-regulated (Table 1) (Supplementary Table S3). Using the Mann–Whitney U Test to study the miRNA expression difference between tumour and control samples, only the difference for EBV-miR-BHRF1-2-5p was statistically significant (P 0.0173), while the difference for miR-222-3p, miR-26b-5p, and EBV-miR-BHRF1-2-3p did not reach the level of significance (P 0.652, P 0.764 and P 0.944 respectively) (Supplementary Fig. S3). BMI1 was positive in 27 samples (67.5%). (Supplementary Figure S1a) (14 mild, 11 moderate, and 2 high) and negative in 13 samples (32.5%) (Supplementary figure S1b). PIM2 showed positivity in 31 samples (77.5%) (Supplementary Figure S2a) (22 mild, 7 moderate, and 2 high) and negativity in 9 samples (22.5%) (Supplementary figure S2b) (Tables 1 and 2).

**Table 1:** Expression profile of the studied markers in DLBCL patients.

Expression results	Number	Percent (%)	Median fold regulation
miR-222-3p			
Up	29	72.5	3 (min. 3–max.141)
Down	11	27.5	− 27.02 (min. − 500 to max. − 2.07)
miR-26b-5p			
Up	29	72.5	2.5 (min. 2.5–max. 110.35)
Down	11	27.5	− 33.33 (min. − 500 to max − 2.439)
ebv-miR-BHRF1-2-5p			
Up	25	62.5	50.861 (min. 2.585–max. 2340.33)
Down	6	15	− 164.04 (min. − 1533.84 to max.− 7.69)
Normal	9	22.5	1.109 (min. − 1.17 to max 1.745)
ebv-miR-BHRF1-2-3p			
Up	27	67.5	3.458 (min. 3.458—max. 13.689)
Down	13	32.5	− 28.49 (min. − 90.09 to max. − 8.19)
BMI-1			
+ Ve	27	67.5	–
− Ve	13	32.5	–
PIM-2			
+ Ve	31	77.5	–
− Ve	9	22.5	–

Diffuse Large B Cell Lymphoma (DLBCL), B lymphoma Mo-MLV insertion region 1 homolog (BMI1), Prodigal insertion site in Maloney murine leukemia virus (PIM).

**Table 2:** Immuohistochemical analysis of the BMI1 and PIM2 according to the intensity and the extent of staining in the bone marrow samples of DLBCL patients.

Marker	BMI1			PIM2		
+ VE	27 (67.5%)			31 (77.5%)		
	Mild (Less than 20%) ( +) 14	Moderate (20–70%) (+ +) 11	High (70–100%) (+ + +) 2	Mild (6–25%) ( +) 22	Moderate (26–75%) (+ +) 7	High (76–100%) (+ + +) 2
− VE	13 (32.5%)			9 (22.5%)		

B lymphoma Mo-MLV insertion region 1 homolog (BMI1), Prodigal insertion site in Maloney murine leukemia virus (PIM), Diffuse Large B Cell Lymphoma (DLBCL).

### Serum miRNAs and the bone marrow proteins are not significant to the clinicopathological parameters

By studying the correlation between the studied miRNA levels and the different clinicopathological parameters, we did not find a significant correlation (Supplementary Table S4). Also, there was no significant correlation between the expression of both bone marrow protein markers (BMI1 and PIM2) and the clinicopathological criteria of the patients (Supplementary Table S5).

### LMR is significantly associated with miR-222-3P and miR-26b-5p levels

When correlating the expression level of the studied miRNA markers to the haematological characteristics, we found a significant correlation between the lymphocyte to monocyte ratio and miR-222-3P (*P* = 0.043) (corrected *p* = 0.55), the lymphocyte to monocyte ratio and miR-26b-5p (*P* = 0.043) (corrected *p* = 0.55), and haemoglobin level with EBV-miR-BHRF1-2-3p (*P* = 0.027) (corrected *p* = 0.648) (Table 3). However, BM BMI1 and PIM2 protein markers did not show any significance with the hematological parameters (Table 4).

**Table 3:** Relation between the studied miRNAs and hematological characteristics of DLBCL patients.

Characteristics	Mir-222-3p		*P* value	Mir-26b-5p		*P* value	ebv-miR-BHRF1-5p			*P* value	ebv-miR-BHRF1-2-3p		*P* Value
	≤ −2	≥ 2		≤ -2	≥ 2		≤ -2	− 1.9–1.9	≥ 2		≤ −2	≥ 2	
Lym/mon													
< 3	2 (11.1%)	14 (77.8%)	0.043 (corrected 0.559)	2 (11.1%)	16 (88.9%)	0.043* (corrected 0.559)	1 (5.6%)	3 (16.7%)	14 (77.8%)	0.202	7 (38.9%)	11 (61.1%)	0.200
≥ 3	8 (40.0%)	15 (68.2%)		8 (40.0%)	12 (60.0%)		4 (20.0%)	6 (30.0%)	10 (50.0%)		4 (20.0%)	16 (80.0%)	
Neut/mon													
< 8	4 (18.2%)	18 (81.8%)	0.142	4 (18.2%)	18 (81.8%)	0.142	3 (13.6%)	3 (13.6%)	16 (72.7%)	0.235	7 (31.8%)	15 (68.2%)	0.736
≥ 8	6 (40.0%)	9 (60.0%)		6 (40.0%)	9 (60.0%)		2 (13.3%)	6 (40.0%)	7 (46.7%)		4 (26.7%)	11 (73.3%)	
Pl/lym													
< 134	6 (30%)	14 (70%)	0.625	6 (30%)	14 (70%)	0.625	6 (31.6%)	3 (15.8%)	10 (52.6%)	0.241	12 (63.2%)	7 (36.8%)	0.812
≥ 134	3 (17.6%)	14 (82.4%)		3 (17.7%)	14 (82.3%)		2 (11.1%)	2 (11.1%)	14 (77.8%)		13 (72.3%)	5 (27.7%)	
Neu/lym													
< 2.131	7 (35%)	13 (65%)	0.177	5 (25%)	15 (75%)	0.861	5 (26.3%)	3 (15.8%)	11 (57.9%)	0.787	14 (73.7%)	5 (26.3%)	0.727
≥ 2.131	2 (11.1%)	16 (88.9%)		5 (27.8%)	13 (72.2%)		4 (21.1%)	2 (10.5%)	13 (68.4%)		12 (63.2%)	7 (36.8%)	
SII													
< 477.189	6 (31.6%)	13 (68.4%)	0.712	6 (30%)	14 (70%)	0.861	4 (21.1%)	2 (10.5%)	13 (68.4%)	0.106	6 (31.6%)	13 (68.4%)	0.727
≥ 477.189	4 (21.1%)	15 (78.9%)		4 (22.2%)	14 (77.8%)		2 (10.5%)	2 (10.5%)	15 (79%)		6 (31.6%)	13 (68.4%)	
Hb													
< 12	6 (28.6%)	15 (71.4%)	0.726	6 (28.6%)	15 (71.4%)	0.726	3 (14.3%)	6 (28.6%)	12 (57.1%)	0.719	3 (14.3%)	18 (85.7%)	0.027* (corrected *p* = 0.648)
≥ 12	4 (23.5%)	13 (76.5%)		4 (23.5%)	13 (76.5%)		2 (11.8%)	3 (17.6%)	12 (70.6%)		8 (47.1%)	9 (52.9%)	
PLT													
< 150	1 (14.3%)	6 (85.7%)	0.424	1 (14.3%)	6 (85.7%)	0.424	0 (0.0%)	2 (28.6%)	5 (71.4%)	0.697	3 (42.9%)	4 (57.1%)	0.390
≥ 150	9 (29.0%)	22 (71.0%)		9 (29.0%)	22 (71.0%)		5 (16.1%)	7 (22.6%)	19 (61.3%)		8 (25.8%)	23 (74.2%)	

Diffuse Large B Cell Lymphoma (DLBCL), Lymphocyte/Monocyte ratio (Lym/mon), Neutrophil monocyte ration (Neut/mon), Platelet lymphocyte ratio (Pl/lym), Neutrophil lymphocyte ration (Neu/lym), Systemic immune-inflammation index (SII), Hemoglobin (Hb), Platelets (PLT), Significant (0.043*).

**Table 4:** Relation between the studied BM protein makers and hematological characteristics of DLBCL patients.

Characteristics	BMI-1		*P* value	PIM-2		*P* value
	−	+		−	+	
Lym/mon						
< 3	7 (38.9%)	11 (61.1%)	0.358	2 (11.1%)	16 (88.9%)	0.130
≥ 3	5 (25%)	15 (75%)		7 (35.0%)	13 (65.0%)	
Neut/mon						
< 8	6 (27.3%)	16 (72.7%)	0.692	4 (18.2%)	18 (81.8%)	0.292
≥ 8	5 (33.3%)	10 (66.7%)		5 (33.3%)	10 (66.7%)	
Pl/lym						
< 134	6 (31.6%)	13 (68.4%)	0.812	7 (36.8%)	12 (63.2%)	0.149
≥ 134	6 (33.3%)	12 (66.7%)		2 (11.1%)	16 (88.9%)	
Neu/lym						
< 2.131	5 (26.3%)	14{73.7%)	0.727	5 (25%)	15 (75%)	
≥ 2.131	7 (36.8%)	12 (63.2%)		4 (22.2%)	14 (77.8%)	1
SII						
< 477.189	6 (31.6%)	13 (68.4%)	0.727	6 (31.6%)	13 (68.4%)	0.445
≥ 477.189	6 (31.6%)	13 (68.4%)		3 (15.8%)	16 (84.2%)	
Hb						
< 12	5 (23.8%)	16 (76.2%)	0.252	6 (28.6%)	15 (71.4%)	0.476
≥ 12	7 (41.2%)	10 (58.8%)		3 (17.6%)	14 (82.4%)	
`PLT						
< 150	3 (42.9%)	4 (57.1%)	0.656	2 (28.6%)	5 (71.4%)	0.736
≥ 150	9 (29.0%)	22 (71.0%)		7 (22.6%)	24 (77.4%)	

Bone Marrow (BM), Diffuse Large B Cell Lymphoma (DLBCL), B lymphoma Mo-MLV insertion region 1 homolog (BMI1), Prodigal insertion site in Maloney murine leukemia virus (PIM). Lymphocyte/Monocyte ratio (Lym/mon), Neutrophil monocyte ration (Neut/mon), Platelet lymphocyte ratio (Pl/lym), Neutrophil lymphocyte ration (Neu/lym), Systemic immune-inflammation index (SII), Hemoglobin (Hb), Platelets (PLT).

### miR-222-3p, miR-26b-5p and ebv-miR-BHRF1-2-5p are significant to each other

Using the ANOVA test, we correlated the selected markers with each other. There was a highly significant correlation between the expression levels of miR-222-3p and miR-26b-5p (*p* = 0.001) (corrected *p* = 0.026), the expression of miR-222-3p and EBV-miR-BHRF1-2-5p (*p* = 0.001) (corrected *p* = 0.026), and the expression of miR-26b-5p and EBV-miR-BHRF1-2-5p (*p* = 0.001) (corrected p = 0.026). This correlation indicated a common mechanism that correlates the 3 miRNAs (Table 5).

**Table 5:** Relation between the studied markers.

Marker	Mir-222-3p		*P* value	miR-26b-5p		*P* value	ebv-miR-BHRF1-2-5p			*P* value
	≤ − 2	≥ 2		≤ − 2	≥ 2		≤ -2	− 1.9–1.9	≥ 2	
miR-222-3p										
≤ -2										
≥ 2										
miR-26b-5p										
≤ -2	11 (100%)	0 (0.0%)	< 0.001*** (corrected 0.026)							
≥ 2	0 (0.0%)	29 (100%)								
ebv-miR-BHRF1-2-5p										
≤ -2	6 (100%)	0 (0.0%)	< 0.001*** (corrected 0.026)	6 (100.0%)	0 (0.0%)	< 0.001*** (corrected 0.026)				
− 1.9–1.9	4 (44.4%)	5 (55.6%)		4 (44.4%)	5 (55.6%)					
≥ 2	1 (4.0%)	24 (96. %)		1 (4.0%)	24 (96.0%)					
ebv-miR-BHRF1-2-3p										
≤ -2	5 (41.7%)	7 (58.3%)	0.189	5 (41.7%)	7 (58.3%)	0.189	2 (16.7%)	4 (33.3%)	6 (50.0%)	0.483
≥ 2	6 (21.4%)	22 (78.6%)		6 (21.4%)	22 (78.6%)		4 (14.3%)	5 (17.9%)	19 (67.9%)	
BMI-1										
− ve	5 (38.5%)	8 (61.5%)	0.281	5 (38.5%)	8 (61.5%)	0.281	3 (23.1%)	1 (7.7%)	9 (69.2%)	0.302
+ ve	6 (22.2%)	21 (77.8%)		6 (22.2%)	21 (77.8%)		3 (11.1%)	8 (29.6%)	16 (59.3%)	
PIM-2										
−ve	4 (44.4%)	5 (55.6%)	0.196	4 (44.4%)	5 (55.6%)	0.196	2 (22.2%)	3 (33.3%)	4 (44.4%)	0.412
+ ve	7 (22.6%)	24 (77.4%)		7 (22.6%)	24 (77.4%)		4 (12.9%)	6 (19.4%)	21 (67.7%)	

Highly significant (< 0.001***), B lymphoma Mo-MLV insertion region 1 homolog (BMI1), Prodigal insertion site in Maloney murine leukemia virus (PIM).

### Serum miRNA and the bone marrow proteins markers could not significantly predict treatment outcome or survival

The expression level of the studied markers was correlated with patient follow-up data. Using Pearson’s Chi-square, we examined the relation between the studied markers and treatment outcome (recurrence, tumour progression, or mortality), but we did not find any significance (Supplementary Table S6). The Kaplan–Meier method was used to study the correlation between the studied markers and survival. None of the studied miRNA or protein markers showed any significant correlation with overall survival (OS), disease-free survival (DFS), or progression-free survival (PFS).

### LMR can predict disease-free survival

**Figure 1:**
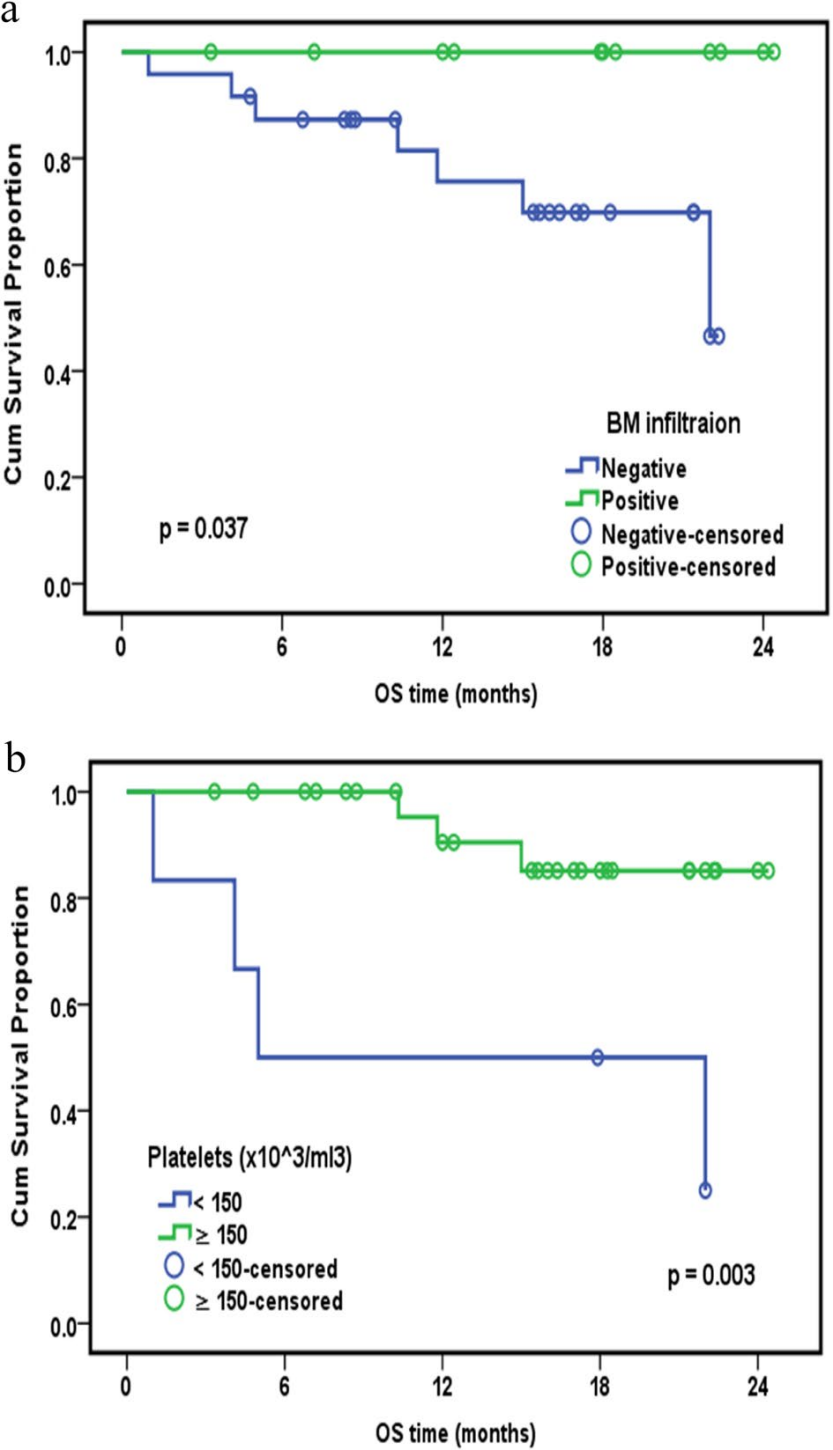
(**a**) Kaplan–Meier curve for OS with BM infiltration. Overall Survival (OS), Bone Marrow infiltration (B M infiltration). (**b**) Kaplan–Meier curve for OS and platelets count. Overall Survival (OS).

**Figure 2:**
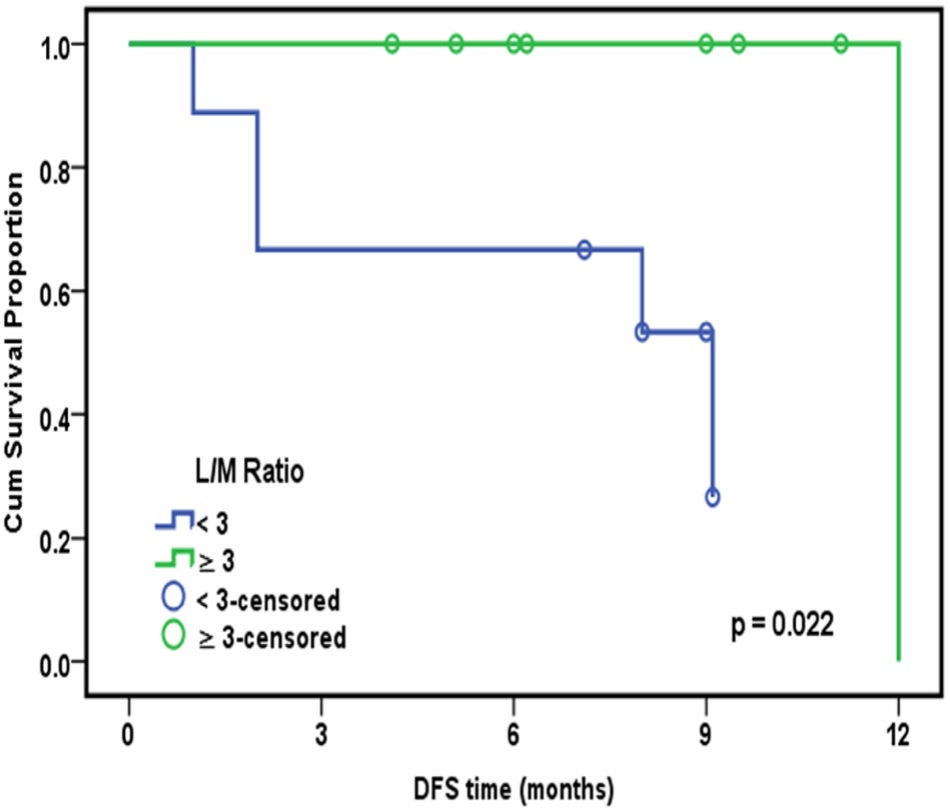
Kaplan–Meier curve for DFS with L/M ratio. Disease Free Survival (DFS), Lymphocyte/Monocyte ratio (L/M ratio).

**Figure 3:**
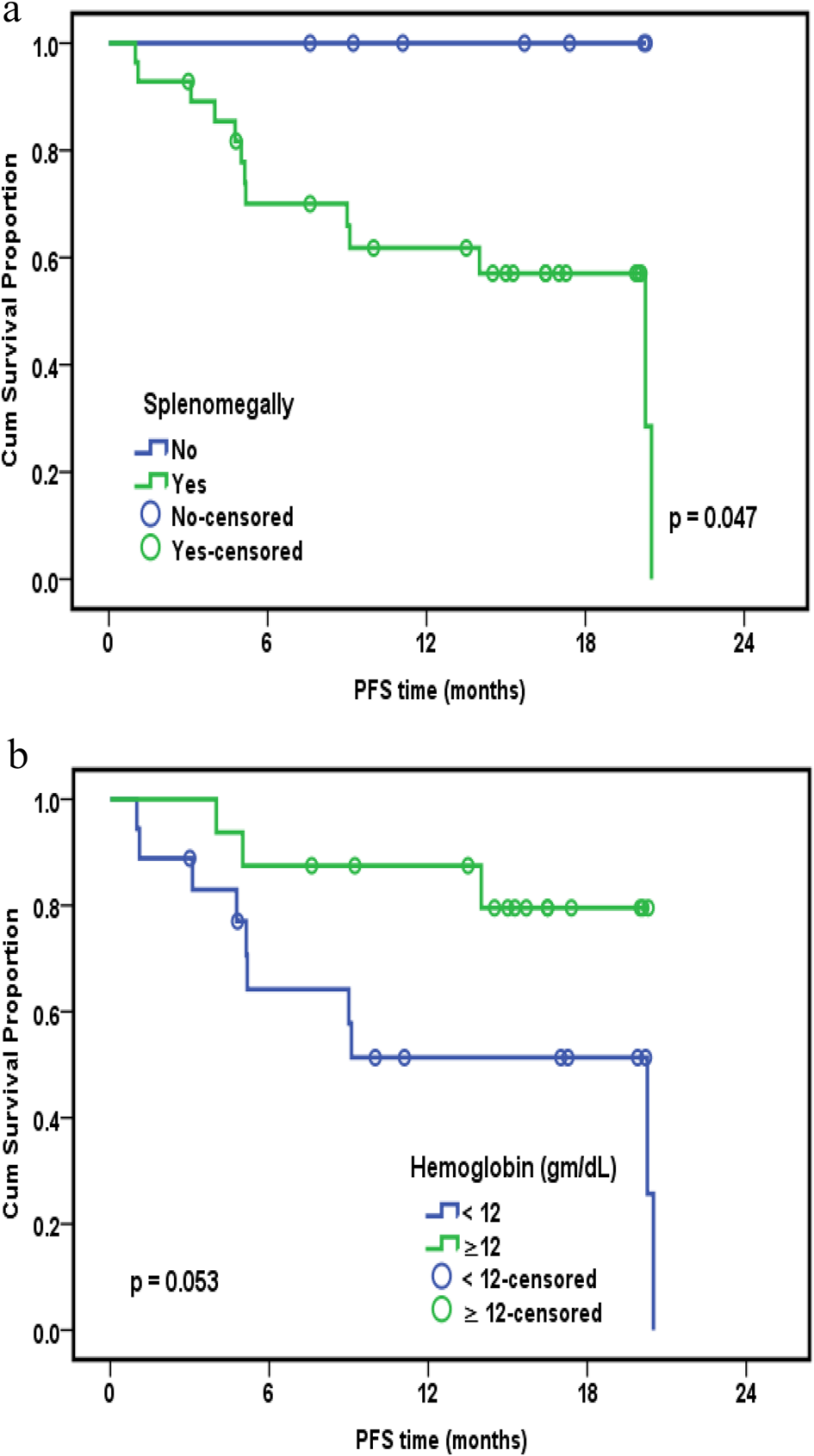
(**a**) Kaplan–Meier curve for PFS with splenomegaly. Progression Free Survival (PFS). (**b**) Kaplan–Meier curve for PFS with hemoglobin level. Progression Free Survival (PFS).

Using the Kaplan–Meier method, we found a significant correlation between overall survival and both BM infiltration (*P* = 0.037) (Fig. 1a) and platelet count (*P* = 0.003) (Fig. 1b). However, overall survival was not significant for the relative lymphocyte to monocyte ratio (*P* = 0.070). Disease-free survival was only significantly correlated to the relative lymphocyte to monocyte ratio (*P* = 0.022) (Fig. 2). However, progression-free survival was significantly correlated with only splenomegaly (*P* = 0.047) (Fig. 3a) and was close to significance with haemoglobin level (*P* = 0.053) (Fig. 3b). Platelet lymphocyte ratio, neutrophil lymphocyte ration and systemic immune-inflammation index did not show any significance with overall survival, disease free survival or progression-free survival.

### LMR, NMR, PLR, NLR and SII are not significant to the clinicopathological parameters

Using Pearson’s Chi-square, we studied the relation between the lymphocyte monocyte ratio, neutrophil monocyte ratio, platelet lymphocyte ratio, neutrophil lymphocyte ratio, and systemic immune-inflammation index with the clinicopathological characteristics of the studied DLBCL patients. We did not find significance between these 5 parameters and any of the patients’ clinicopathological characteristics (Supplementary Table S7).

### LMR, NMR, PLR, NLR and SII did not significantly predict patients’ outcome

By studying the correlation between LMR, NMR, PLR, NLR, and SII ratios and patient outcome data, including treatment outcome, recurrence, tumour progression, and mortality, we could not find any significant correlation (Supplementary Table S8).

### In silico analysis of target gene prediction and pathway enrichment analysis for miR-222-3P, miR-26b-5P and ebv-miR-BHRF-1-2-5P revealed common genes and pathways involved in immunological and pathogenesis mechanisms

In silico analysis was applied to detect the potential target genes for the significantly associated miRNAs; miR-222-3p, miR-26b-5p and ebv-miR-BHRF-1-2-5p using DIANA-TarBase v8. Analysis revealed 545 target genes for miR-222-3p, 2688 target genes for miR-26b-5p and 137 target genes for ebv-miR-BHRF-1-2-5p (supplementary table S9). Using Venn diagram, we found 166 overlapping target genes between miR-222-3p and miR-26b-5p and 9 overlapping target genes between the three miRNAs (Supplementary figure S4a).

Functional enrichment analysis using Metascape database showed that the 166 genes are enriched in GO Biological processes and reactome gene sets including cytokine signaling in immune system, hematopoiesis, definitive hemopoiesis, G1 and G1/S transition, metabolism, apoptosis, cyclin B, hormones and post translation modification (Supplementary figure S4b) while the 9 target genes were mainly enriched in lymphoma and leukemia pathogenesis (Supplementary figure S4c).

The results of analysis showed that the common target genes and pathways between the 3 miRNAs are involved in DLBCL pathogenesis, while the common target genes and pathways between miR-222-3P, and miR-26b-5P are involved in cytokine signaling in immune system. This may indicates that the influence of the 3 miRNAs on DLBCL pathogenesis may exist. The analysis also indicated the possible influence of both miR-222-3P, and miR-26b-5P on immunological mechanisms that may indirectly affect LMR in DLBCL.

## Discussion

DLBCL is the most common subtype of NHL in Egypt, representing about 49% of all NHL cases presented to the NCI^43^.

Different genes were studied and showed prognostic significance in DLBCL, such as TP53^44^, MYC, and BCL2^45^. Other markers that showed a promising prognostic significance are CD44^46^, survivin^47^, NF-κB, p65, Stat3, Syk, BTK, Pax5, Bcl6, and P57KIP2^48^.

Most of the above-mentioned molecular markers have been studied on tumour biopsies or from bone marrow samples of patients, and this requires invasive techniques to collect samples. In addition, the correlations between these markers and the clinical and prognostic data of the patients are contradictory. This requires using minimally invasive or non-invasive techniques with high accuracy for the diagnosis and prognosis of DLBCL. Among these techniques is the use of blood or urine samples from the patients.

Recent studies showed that epigenetic abnormalities are hallmarks of cancer and occur early in the carcinogenesis process^49^. miRNA is an important epigenetic factor in regulating gene expression and, accordingly, cell fate. This notion is important in cancer, where the regulation of gene expression can influence the transformation status of normal cells.

Our results indicated dysregulation of miR-222-3p in all studied DLBCL cases. Other studies detected miR-222-3p dysregulation in DLBCL^14^. Also, miR-222-3p was shown to be dysregulated in other tumors, such as gastric cancer^50^ and breast cancer^51^. We did not find significance between miR-222-3p expression and any of the patient’s clinical or follow-up data. However, previous data regarding this point are contradictory where some data showed significance^52^ and others did not show significance^53^.

Interestingly, miR-222-3p showed significant correlation with LMR. Studies regarding LMR in cancer found an important role of this ratio on patients’ prognosis where decreased LMR led to low antitumor immunity and accordingly bad prognosis^54^. However, the literature did not register any correlation between serum miRNA and LMR in cancer patients. But miR-222-3p was found to be regulated by interleukins and interferons that regulate immunity ^31^. Other studies found that miR-222-3p can modulate the function of immune cells, such as natural killer (NK) cells, and serve an important role in the tumour microenvironment^32^. This means that the expression of miR-222-3p may interact with or be influenced by several immune factors that affect the patient’s immunity, which in turn affects LMR.

MiR-26b-5p was also dysregulated in all the studied DLBCL cases, with 27.5% of the cases downregulated and 72.5% upregulated. We did not find any study that correlates miR-26b-5p with DLBCL; only one paper studied the role of miR-26b-5p in Burkitt lymphoma; they found a tumour suppressive role for miR-26b-5p where it inhibited BL growth^19^. In contrast to our results in DLBCL, miR-26b-5p expression in solid tumours showed downregulation^17^. We also did not find any significance between miR-26b-5p and the clinical data of our studied cases. Our study is consistent with previous studies, which indicated no significance between miR-26b-5p and clinical data including age, sex, tumour stage^55^, grade, and survival^16^. On the contrary, other studies showed a significant correlation between miR-26b-5p and both survival in HCC^18^ and clinical stage in thyroid cancer^17^.

Similar to miR-222-3p, mir-26b-5p was significantly associated with LMR. This may also reflect the role of this miRNA in patient immunity. It was found that mir-26b-5p triggered T cell responses by targeting PIM-2 in HCC^34^. But miR-26b-5p expression is not enough to induce an immune response against HCC, where immune competence is required for the miR-26b-5p-mediated effect of HCC treatment^34^. This may explain why miR-26b-5p did not significantly affect the survival of the patients in our study, although it is upregulated in most cases. This is because miR-26b-5p may need immune competence to positively affect the patient’s survival.

Accordingly, both of these miRNAs seem to have a common denominator and may play an immune-related role in DLBCL patients. In other words, both miRNAs may be directly or indirectly involved in an immunological mechanism that affects the patient's immunity and influences the LMR, which in turn affects the survival of DLBCL patients.

For this reason we did in silico analysis to study the common target genes and pathways between miR-222-3p and miR-26b-5p. The results of the analysis indicated the implication of both miRNAs in important immunological pathways that may affect the patients’ immunity which may have an impact on LMR.

This result indicates the possibility of a relationship between these 2 miRNAs and the LMR. Accordingly, we need an extensive experimental study that deciphers the relation between these two miRNAs and the immune pathways in DLBCL patients, especially the cytokine signalling in the immune system. This may show how these 2 miRNAs affect the immune mechanism in DLBCL patients. Thus, it may lead to the development of a treatment method or immunotherapy for DLBCL patients.

Although the Chi-Square statistical test demonstrated the existence of a significant relationship between the two miRNAs and the LMR of the patients, the multiple testing correction method nullified this significance. Fortunately, the in silico analysis did confirm the possibility of a relationship between these two miRNAs and an immunological mechanism that may affect LMR in patients, especially since previous studies have shown that low LMR leads to low antitumor immunity^54^.

Our study demonstrated up and downregulation of EBV-miR-BHRF1-2 in serum where EBV-miR-BHRF1-2-5p showed 77.5% dysregulation and EBV-miR-BHRF1-2-3p showed 100% dysregulation. Previous studies did not demonstrate the presence of the EBV-miR-BHRF1 cluster in DLBCL^56^. However, EBV-miR-BHRF1-2-5p was found to be upregulated in other diseases, like multiple sclerosis^57^. This is the first study that correlates the expression of EBV-miR-BHRF1-2-5p and 3p expressions with the clinicopathological and survival data in cancer patients, especially in DLBCL. Although we found a near-significant correlation between EBV-miR-BHRF1-2-5p and disease-free survival, we cannot confirm this relation due to the borderline significance.

BMI1 was expressed in 27 out of 40 bone marrow samples (67.5%). Previous studies detected BMI1 in the tissue of DLBCL^58^ and FL^59^. Unfortunately, we did not find any study that determined BMI1 in the bone marrow of DLBCL patients; actually, we studied BMI1 in bone marrow samples to compare the expression of this marker in BM infiltrative versus non-infiltrative cases. We did not find a significant difference in the expression of this marker between both groups. Also, we did not find any significance between the BM expression of BMI1 and the clinical and survival data. On the other hand, one study demonstrated the significance of BMI1 in the survival of DLBCL patients^58^. The same study did not find any significance between BMI1 and the clinical data of the patients^58^. In follicular lymphoma, BMI1 was significantly correlated to survival^59^. The difference in significance between our results and the results of the other studies may be related to the difference in tissue type, where we used bone marrow samples to detect BMI1 expression while the other studies used tumour tissue biopsies to detect this marker.

Regarding PIM2, 77.5% of BM samples were positive for this marker. Our results are consistent with previous studies that demonstrated the expression of PIM2 in DLBCL^28,60^. The same as BMI1, PIM2 expression did not show any significant correlation with the clinical and survival data. Data from the literature showed the opposite of our findings, where PIM2 showed a significant correlation with the clinicopathological and survival data in DLBCL^28^. This difference in the significance may be related to the tissue type where we used BM samples to detect PIM2 expression, unlike the other studies that used lymph node samples.

According to our data, we may conclude that studying the expression of BMI1 and PIM2 in the bone marrow of DLBCL patients failed to significantly differentiate between BM infiltrative and non-infiltrative patients. In addition, it showed no significance with neither clinical nor follow-up data in DLBCL.

Interestingly, our study showed a highly significant correlation between miR-222-3p, miR-26b-5p, and EBV-miR-BHRF1-2-5p. This correlation may indicate the presence of common target genes and/or pathways between the 3 miRNAs, especially those pathways involved in DLBCL pathogenesis. The results of the in silico analysis for the three miRNAs demonstrated the presence of 9 overlapping target genes directly related to lymphoma and leukemia pathogenesis, which indicate the possible role of these miRNAs in DLBCL pathogenesis. Accordingly, more studies are needed to detect the exact mechanism of DLBCL carcinogenesis by these 3 miRNAs and how we can use the results for treating DLBCL patients.

LMR was significantly correlated with survival. This result indicates a simple predictive method for patient prognosis in DLBCL. Previous studies indicated a prognostic significance of the L/M ratio in DLBCL^61^, but the exact mechanism is not clear.

From the data of our study, we may conclude that our studied markers are important in maintaining the transformation status (malignant phenotype) in DLBCL without influencing the clinical or survival data of the patients. Another suggestion is that these markers may indirectly affect DLBCL by controlling the gene or pathway(s) that influence the progression and survival of DLBCL. The significant correlation between LMR and Mir-222-3p and Mir-26b-5p may indicate the implication of these 2 miRNAs in an immunological mechanism that affects patient’s immunity and accordingly influence LMR. The significant correlation between miR-222-3p, miR-26b-5p, and EBV-miR-BHRF1-2-5p may underscore a common hidden mechanism or pathway(s) between the 3 miRNAs that may explain DLBCL pathogenesis. Targeting our studied markers is needed to examine the potential of these markers in the prevention and treatment of DLBCL. More in-depth studies are needed to exactly detect the role of EBV-miR-BHRF1-2-5p in the survival of DLBCL patients. Serum is a useful, noninvasive alternative method for miRNA detection in DLBCL.

## Supplementary Information

Supplementary Information 1.

Supplementary Information 2.

Supplementary Information 3.

Supplementary Information 4.

Supplementary Information 5.

Supplementary Information 6.

Supplementary Information 7.

Supplementary Information 8.

Supplementary Information 9.

Supplementary Information 10.

Supplementary Information 11.

Supplementary Information 12.

Supplementary Information 13.

## Data Availability

The datasets generated during and/or analyzed during the current study are available in the manuscript.
