# Supplementary material for: Lymphocyte to monocyte ratio predicts survival and is epigenetically linked to miR-222-3p and miR-26b-5p in diffuse large B cell lymphoma
Source: Sci Rep. 2023 Mar 25;13:4899. doi: 10.1038/s41598-023-31700-x (PMC10039925; doi:10.1038/s41598-023-31700-x)
Supplement: Supplementary file 1 — Supplementary Information 1. [file 41598_2023_31700_MOESM1_ESM.docx]

**Supplementary Table (S1): The Clinico-pathological characteristics of the studied DLBCL patients.**

| **Patient Characteristics** | | **Number** | **Percent** |
| --- | --- | --- | --- |
| **Median Age** | 51.5 (20.0 – 80.0) | 40 | 100% |
| **Sex** | Male | 19 | 47% |
|  | Female | 21 | 53 % |
| **Clinical stage** | I | 1 | 2.5 % |
|  | II | 6 | 15 % |
|  | III | 14 | 35% |
|  | IV | 16 | 40% |
|  | ND | 3 | 7.5% |
| **Splenomegaly** | Yes | 32 | 80 % |
|  | No | 7 | 7.5 % |
|  | ND | 1 | 2.5% |
| **B M infiltration** | Yes | 26 | 65 % |
|  | No | 14 | 35% |
| **Reticulin** | Positive | 12 | 30 % |
|  | Negative | 15 | 37.5% |
|  | ND | 13 | 32.5% |
| **B symptoms** | Positive | 17 | 42.5 % |
|  | Negative | 22 | 55 % |
|  | ND | 1 | 2.5% |
| **Median LDH** | 405.5 (171.0 – 2099) | 9 | 40.9 % |
| **Median B2M** | 3.06 (2.34 – 8.80) | 4 | 18.2% |
| **HCV** | Positive | 14 | 35% |
|  | Negative | 20 | 50% |
|  | ND | 6 | 15% |
| **HbsAg** | Positive | 1 | 2.5% |
|  | Negative | 33 | 82.5% |
|  | ND | 6 | 15% |
| **HIV** | Positive | 0 | 0% |
|  | Negative | 34 | 85% |
|  | ND | 6 | 15% |

Diffuse Large B Cell Lymphoma (DLBCL), Bone Marrow infiltration (B M infiltration), Lactate Dehydrogenase (LDH), Beta 2 Microglobulin (B2M), Hepatitis C Virus (HCV), Hepatitis B Surface Antigen ( HbsAg), Human Immunodificiency Virus (HIV).
