# Supplementary material for: Lymphocyte to monocyte ratio predicts survival and is epigenetically linked to miR-222-3p and miR-26b-5p in diffuse large B cell lymphoma
Source: Sci Rep. 2023 Mar 25;13:4899. doi: 10.1038/s41598-023-31700-x (PMC10039925; doi:10.1038/s41598-023-31700-x)
Supplement: Supplementary file 2 — Supplementary Information 2. [file 41598_2023_31700_MOESM2_ESM.docx]

**Supplementary Table (S2): Follow up data results of the studied DLBCL patients.**

| **Follow up data** | | **Number** | **Percent** |
| --- | --- | --- | --- |
| **Chemotherapy** | Yes | 35 | 87.5 % |
|  | No | 5 | 12.5 % |
| **Chemotherapy outcome** | Sensitive | 17 | 48.6 % |
|  | Resistance | 12 | 34.3 % |
|  | Partial remission | 4 | 11.4% |
|  | Incomplete therapy | 2 | 5.7% |
| **Recurrence** | Yes | 5 | 29.4% |
|  | No | 12 | 70.6% |
| **Progression** | Yes | 7 | 58.3% |
|  | No | 5 | 41.7% |
| **Median follow up/month** | 12.1 (0.1-24.1) | 40 | 100 % |
| **Median DFS/ month** | 8 (2- 22), | 40 | 100% |
| **Median PFS/month** | 8 (1.1-20.27) | 40 | 100% |
| **Median OS/month** | 15 (0.06- 24.4). | 40 | 100% |

Diffuse Large B Cell Lymphoma (DLBCL), Disease Free Survival (DFS), Progression Free Survival (PFS), Overall Survival (OS).
