# Supplementary material for: Lymphocyte to monocyte ratio predicts survival and is epigenetically linked to miR-222-3p and miR-26b-5p in diffuse large B cell lymphoma
Source: Sci Rep. 2023 Mar 25;13:4899. doi: 10.1038/s41598-023-31700-x (PMC10039925; doi:10.1038/s41598-023-31700-x)
Supplement: Supplementary file 3 — Supplementary Information 3. [file 41598_2023_31700_MOESM3_ESM.docx]

**Supplementary table (S3): Fold change data of the studied micro RNAs.**

| **Sample number** | **miR-222-3p** | **mir-26b-5p** | **EBV-miR-BHRF1-2-5p** | **EBV-miR-BHRF1-2-3p** |
| --- | --- | --- | --- | --- |
| 1 | 3 | 2.5 | 1.348 | 3.458 |
| 2 | 3 | 2.5 | 14.2165 | 3.458 |
| 3 | 3 | 2.5 | 54.285 | 3.458 |
| 4 | 3 | 2.5 | 1.193 | 3.458 |
| 5 | 3 | 2.5 | 0.936 | 3.458 |
| 6 | 0.009 | 0.0079 | 0.0065 | 3.458 |
| 7 | 0.037 | 0.03 | 0.0236 | 3.458 |
| 8 | 0.002 | 0.002 | 0.00119 | 3.458 |
| 9 | 3 | 2.5 | 31.922 | 0.053 |
| 10 | 0.006 | 0.005 | 0.0035 | 3.458 |
| 11 | 3 | 2.5 | 71.283 | 0.11 |
| 12 | 3 | 2.5 | 1.745 | 0.0456 |
| 13 | 3 | 2.5 | 15.632 | 3.458 |
| 14 | 3 | 2.5 | 0.96 | 3.458 |
| 15 | 3 | 2.5 | 76.665 | 0.0213 |
| 16 | 0.111 | 0.092 | 199.2588 | 0.0754 |
| 17 | 3 | 2.5 | 2.585 | 3.458 |
| 18 | 3 | 2.5 | 4.895 | 3.458 |
| 19 | 3 | 2.5 | 69.43 | 3.458 |
| 20 | 3 | 2.5 | 86.612 | 3.458 |
| 21 | 3 | 2.5 | 425.348 | 0.0182 |
| 22 | 3 | 2.5 | 5.838 | 3.458 |
| 23 | 3 | 2.5 | 1122.5 | 0.0271 |
| 24 | 3 | 2.5 | 2340.336 | 0.0351 |
| 25 | 3 | 2.5 | 3.867 | 3.458 |
| 26 | 0.036 | 0.03 | 1.109 | 0.042 |
| 27 | 3 | 2.5 | 17.578 | 3.458 |
| 28 | 0.245 | 0.41 | 0.028 | 0.0111 |
| 29 | 141.141 | 2.5 | 45.743 | 3.458 |
| 30 | 13.122 | 2.5 | 129.831 | 3.458 |
| 31 | 7.1 | 2.5 | 50.861 | 3.458 |
| 32 | 10.882 | 2.5 | 31.006 | 13.689 |
| 33 | 0.078 | 0.065 | 0.958 | 4.187 |
| 34 | 3 | 110.35 | 20.771 | 3.458 |
| 35 | 0.106 | 0.088 | 0.851 | 0.122 |
| 36 | 3 | 2.5 | 32.055 | 3.458 |
| 37 | 0.481 | 0.019 | 0.13 | 0.0265 |
| 38 | 3 | 20.08 | 379.064 | 3.458 |
| 39 | 3 | 2.5 | 120.634 | 3.458 |
| 40 | 0.023 | 0.08 | 1.574 | 0.0264 |
