# Supplementary material for: Lymphocyte to monocyte ratio predicts survival and is epigenetically linked to miR-222-3p and miR-26b-5p in diffuse large B cell lymphoma
Source: Sci Rep. 2023 Mar 25;13:4899. doi: 10.1038/s41598-023-31700-x (PMC10039925; doi:10.1038/s41598-023-31700-x)
Supplement: Supplementary file 4 — Supplementary Information 4. [file 41598_2023_31700_MOESM4_ESM.docx]

**Supplementary Table (S4): Relation between the expression of the studied serum miRNAs and the clinicopathological characteristics of DLBCL patients.**

| **Characteristics** | **Mir-222-3p** | | **P value** | **Mir-26b-5p** | | **P value** | **ebv-miR-BHRF1-5p** | | | **P value** | **ebv-miR-BHRF1-2-3p** | | **P Value** |
| --- | --- | --- | --- | --- | --- | --- | --- | --- | --- | --- | --- | --- | --- |
|  | **=< -2** | **>=2** |  | **≤ -2** | **≥ 2** |  | **≤ -2** | **-1.9–1.9** | **≥ 2** |  | **≤-2** | **≥ 2** |  |
| **Age**  **< 50 y**  **>= 5 0y** | 4(22.2%)  7(31.8%) | 14(77.8%)  15(68.2%) | 0.499 | 4(22.2%)  7(31.8%) | 14(77.8%  15(68.2%) | 0.499 | 3(16.7%)  3(13.6%) | 3(16.7%)  6(27.3%) | 12(66.7%)  13(59.1%) | 0.814 | 3(16.7%)  9(40.9%) | 15(83.3%)  13(59.1%) | 0.096 |
| **Gender**  **Male**  **Female** | 3(15.8%)  8(38.1%) | 16(84.2)  13(61.9) | 0.115 | 3(15.8%)  8(38.1%) | 16(84.2%)  13(61.9%) | 0.115 | 2(10.5%)  4(19.0%) | 4(21.1%)  5(23.8%) | 13(68.4%)  12(57.1%) | 0.815 | 7(36.8%)  5(23.8%) | 12(63.2%)  16(76.2%) | 0.369 |
| **Stage**  **I, II**  **III**  **IV** | 2(28.6%)  3(21.4%)  6(37.5%) | 5(71.4%)  11(78.6%)  10(62.5%) | 0.729 | 2(28.6%)  3(21.4%)  6(37.5%) | 5(71.4%)  11(78.6%)  10(62.5%) | 0.729 | 1(14.3%)  1(7.1%)  4(25.0%) | 0(0.0%)  6(42.9%)  3(18.8%) | 6(85.7%)  7(50.0%)  9(56.3%) | 0.194 | 2(28.6%)  4(28.6%)  4(25.0%) | 5(71.4%)  10(71.4%)  12(75.0%) | 1.000 |
| **LDH**  **< 400**  **>= 400** | 7(35.0%)  4(20.0%) | 13(65.0%)  16(80.0%) | 0.288 | 7(35.0%)  4(20.0%) | 13(65.0%)  16(80.0%) | 0.288 | 3(15.0%)  3(15.0%) | 5(25.0%)  4(20.0%) | 12(60.0%)  13(65.0%) | 1.000 | 7(35.0%)  5(25.0%) | 13(65.0%)  15(75.0%) | 0.490 |
| **B2M**  **< 4**  **>= 4** | 3(30.0%)  4(57.1%) | 7(70.0%)  3(42.9%) | 0.350 | 3(30.0%)  4(57.1%) | 7(70.0%)  3(42.9%) | 0.350 | 0(0.0%)  3(42.9%) | 2(20.0%)  1(14.3%) | 8(80.0%)  3(42.9%) | 0.119 | 5(50.0%)  2(28.6%) | 5(50.0%)  5(71.4%) | 0.622 |
| **HCV**  **-ve**  **+ve** | 5(26.3%)  3(20.0%) | 14(73.7%)  12(80.0%) | 1.000 | 5(26.3%)  3(20.0%) | 14(73.7%)  12(80.0%) | 1.000 | 1(5.3%)  2(13.3%) | 6(31.6%)  2(13.3%) | 12(63.2%)  11(73.3%) | 0.478 | 6(31.6%)  4(26.7%) | 13(68.4%)  11(73.3%) | 0.755 |
| **Splenomegaly**  **-ve**  **+ve** | 3(42.9%)  7(21.9%) | 4(57.1%)  25(78.1%) | 0.249 | 3(42.9%)  7(21.9%) | 4(57.1%)  25(78.1%) | 0.249 | 1(14.3%)  4(12.5%) | 2(28.6%)  7(21.9%) | 4(57.1%)  21(65.6%) | 0.845 | 2(28.6%)  9(28.1%) | 5(71.4%)  23(71.9%) | 0.981 |
| **Bsymptoms**  **-ve**  **+ve** | 7(31.8%)  3(17.6%) | 15(68.2%)  14(82.4%) | 0.315 | 7(31.8%)  3(17.6%) | 15(68.2%)  14(82.4%) | 0.315 | 4(18.2%)  1(5.9%) | 6(27.3%)  3(17.6%) | 12(54.5%)  13(76.5%) | 0.400 | 7(31.8%)  4(23.5%) | 15(68.2%)  13(76.5%) | 0.568 |
| **Reticulin**  **-ve**  **+ve** | 5(26.3%)  5(29.4%) | 14(73.7%)  12(70.6%) | 0.836 | 5(26.3%)  5(29.4%) | 14(73.7%)  12(70.6%) | 0.836 | 3(15.8%)  3(17.6%) | 3(15.8%)  6(35.3%) | 13(68.4%)  8(47.1%) | 0.339 | 5(26.3%)  6(35.3%) | 14(73.7%)  11(64.7%) | 0.559 |
| **BM infiltrat.**  **-ve**  **+ve** | 7(26.9%)  4(28.6%) | 19(73.1%)  10(71.4%) | 0.911 | 7(26.9%)  4(28.6%) | 19(73.1%)  10(71.4%) | 0.911 | 3(11.5%)  3(21.4%) | 5(19.2%)  4(28.6%) | 18(69.2%)  7(50.0%) | 0.446 | 10(38.5%)  2(14.3%) | 16(61.5%)  12(85.7%) | 0.112 |

Diffuse Large B Cell Lymphoma (DLBCL), micro RNA (miRNAs), Bone Marrow infiltration (B M infiltration), Lactate Dehydrogenase (LDH), Beta 2 Microglobulin (B2M), Hepatitis C Virus (HCV).
