# Supplementary material for: Lymphocyte to monocyte ratio predicts survival and is epigenetically linked to miR-222-3p and miR-26b-5p in diffuse large B cell lymphoma
Source: Sci Rep. 2023 Mar 25;13:4899. doi: 10.1038/s41598-023-31700-x (PMC10039925; doi:10.1038/s41598-023-31700-x)
Supplement: Supplementary file 5 — Supplementary Information 5. [file 41598_2023_31700_MOESM5_ESM.docx]

**Supplementary Table (S5): Relation between the expression of the studied BM protein markers and the clinicopathological characteristics of DLBCL patients.**

| **Characteristics** | **BMI-1** | | **P value** | **PIM-2** | | **P value** |
| --- | --- | --- | --- | --- | --- | --- |
|  | **-** | **+** |  | **-** | **+** |  |
| **Age**  **< 50 y**  **>= 5 0y** | 7(38.9%)  6(27.3%) | 11 (50%)  16(72.7%) | 0.435 | 4(22.2%)  5 22.7%) | 14(77.8%)  17(77.3%) | 1.000 |
| **Gender**  **Male**  **Female** | 5(26.3%)  8(38.1%) | 14(73.7%)  13(61.9%) | 0.427 | 4(21.1%)  5(23.8%) | 15(78.9%)  16(76.2%) | 1.000 |
| **Stage**  **I, II**  **III**  **IV** | 2(28.6%)  2(14.3%)  6(37.5%) | 5(71.4%)  12(85.7%)  10(62.5%) | 0.384 | 1(14.3%)  3(21.4%)  5(31.3%) | 6(85.7%)  11(78.6%)  11(68.8%) | 0.691 |
| **LDH**  **< 400**  **>= 400** | 8(40.0%)  5(25.0%) | 12(60.0%)  15(75.0%) | 0.311 | 6(30.0%)  3(15.0%) | 14(70.0%)  17(85.0%) | 0.451 |
| **B2M**  **< 4**  **>= 4** | 3(30.0%)  2(28.6%) | 7(70.0%)  5(71.4%) | 1.000 | 3(30.0%)  2(28.6%) | 7(70.0%)  5(71.4%) | 1.000 |
| **HCV**  **-ve**  **+ve** | 4(21.1%)  7(46.7%) | 15(78.9%)  8(53.3%) | 0.113 | 5(26.3%)  4(26.7%) | 14(73.7%)  11(73.3%) | 0.982 |
| **Splenomegaly**  **-ve**  **+ve** | 3(42.9%)  10(31.3%) | 4(57.1%)  22(68.8%) | 0.666 | 3(42.9%)  6(18.8%) | 4(57.1%)  26(81.3%) | 0.170 |
| **Bsymptoms**  **-ve**  **+ve** | 6(27.3%)  7(41.2%) | 16(72.7%)  10(58.8%) | 0.361 | 7(31.8%)  2(11.8%) | 15(68.2%)  15(88.2%) | 0.141 |
| **Reticulin**  **-ve**  **+ve** | 7(36.8%)  3(17.6%) | 12(63.2%)  14(82.4%) | 0.199 | 6(31.6%)  3(17.6%) | 13(68.4%)  14(82.4%) | 0.451 |
| **BM infiltration**  **-ve**  **+ve** | 10(38.5%)  3(21.4%) | 16(61.5%)  11(78.6%) | 0.273 | 6(23.1%)  3(21.4%) | 20(76.9%)  11(78.6%) | 0.905 |

Bone Marrow (B M), Diffuse Large B Cell Lymphoma (DLBCL), Lactate Dehydrogenase (LDH), Beta 2 Microglobulin (B2M), Hepatitis C Virus (HCV).
