# Supplementary material for: Lymphocyte to monocyte ratio predicts survival and is epigenetically linked to miR-222-3p and miR-26b-5p in diffuse large B cell lymphoma
Source: Sci Rep. 2023 Mar 25;13:4899. doi: 10.1038/s41598-023-31700-x (PMC10039925; doi:10.1038/s41598-023-31700-x)
Supplement: Supplementary file 6 — Supplementary Information 6. [file 41598_2023_31700_MOESM6_ESM.docx]

**Supplementary Table (S6): Relation between the studied markers and, treatment outcome, tumor recurrence, tumor progression and mortality in the studied DLBCL.**

| **Markers** | **Treatment outcome** | | **P value** | **Tumor recurrence** | | **P value** | **Tumor progression** | | **P value** | **Mortality** | | **P value** |
| --- | --- | --- | --- | --- | --- | --- | --- | --- | --- | --- | --- | --- |
|  | **Sensitive + Partial**  **(n = 21)** | **Resistant**  **(n = 12)** |  | **Recurrent**  **(n = 4)** | **Non-recurrent**  **(n = 13)** |  | **Progressive**  **(n = 7)** | **Non-progressive**  **(n = 5)** |  | **Died**  **(n = 7)** | **Alive (n = 29)** |  |
| **Mir-222-3p**  **=< -2**  **>=2** | 7(33.3%)  14(66.7%) | 1 (8.3%)  11(91.7%) | 0.107 | 0 (0%)  4(23.5%) | 5(29.4%)  8(47.1%) | 0.2605 | 1 (14.3%)  6 (85.7%) | 0 (0%)  5 (100%) | 1 | 2(14.3%)  5(85.7%) | 7 (0%)  22(100%) | 1 |
| **Mir-26b-5p**  **=< -2**  **>=2** | 7(33.3%)  14(66.7%) | 1 (8.3%)  11(91.7%) | 0.107 | 0(0%)  4(23.5%) | 5(29.4%)  8(47.1%) | 0.2605 | 1 (14.3%)  6 (85.7%) | 0 (0%)  5 (100%) | 1 | 2(14.3%)  5(85.7%) | 7 (0%)  22(100%) | 1 |
| **ebv-miR-BHRF1-2-5p**  **=< -2**  **-1.9-1.9**  **>=2** | 4(19.0%)  4(19.0%)  13(61.9%) | 1 (8.3%)  2(16.7%)  9(75.0%) | 0.865 | 0(14.3%)  4(71.4%) | 6(20%)  7(80%) | 0.237 | 2 (14.3%)  5 (71.4%) | 1 (20%)  4 (80%) | 1 | 2(14.3%)  5(71.4%) | 10 (20%)  19 (80%) | 1 |
| **ebv-miR-BHRF1-2-3p**  **=< -2**  **>=2** | 7(33.3%)  14(66.7%) | 2(16.7%)  10(83.3%) | 0.301 | 1(33.3%) 3(66.7%) | 5 (40%)  8 (60%) | 1 | 0 (33.3%)  7 (66.7%) | 2 (40%)  3 (60%) | 0.151 | 2(33.3%)  5(66.7%) | 8 (40%)  21 (60%) | 1 |
| **BMI-1**  **-ve**  **+ve** | 8(38.1%)  13(61.9%) | 4(33.3%)  8(66.7%) | 0.784 | (14.3%)  (85.7%) | (33.3%)  (66.7%) | 0.537 | 1 (14.3%)  6 (85.7%) | 3 (33.3%)  2 (66.7%) | 0.222 | 3(14.3%)  4(85.7%) | 9 (33.3%)  20 (66.7) | 0.225 |
| **PIM-2**  **-ve**  **+ve** | 4(19.0%)  17(81.0%) | 4(33.3%)  8(66.7%) | 0.357 | (43%)  (57%) | (20%)  (80%) | 0.541 | 3 (43%)  4 (57%) | 1 (20%)  4 (80%) | 0.575 | 3 (43%)  4 (57%) | 6 (20%)  23 (80%) | 0.332 |

Diffuse Large B Cell Lymphoma (DLBCL), Number (n), B lymphoma Mo-MLV insertion region 1 homolog (BMI1), Prodigal insertion site in Maloney murine leukemia virus (PIM).
