# Supplementary material for: Lymphocyte to monocyte ratio predicts survival and is epigenetically linked to miR-222-3p and miR-26b-5p in diffuse large B cell lymphoma
Source: Sci Rep. 2023 Mar 25;13:4899. doi: 10.1038/s41598-023-31700-x (PMC10039925; doi:10.1038/s41598-023-31700-x)
Supplement: Supplementary file 7 — Supplementary Information 7. [file 41598_2023_31700_MOESM7_ESM.docx]

**Supplementary Table (S7): Relation between LMR, NMR, PLR, NLR, SII and the clinicopathological characteristics of DLBCL patients.**

| **Characteristics** | **Lym/mon** | | **P value** | **Neu/mon** | | **P value** | **Pl/lym** | | **P value** | **Neu/lym** | | **P Value** | **SII** | | **P Value** |
| --- | --- | --- | --- | --- | --- | --- | --- | --- | --- | --- | --- | --- | --- | --- | --- |
|  | **<3** | **≥ 3** |  | **< 7.4** | **≥ 7.4** |  | **< 134.273** | **≥134.273** |  | **<2.131** | **≥ 2.131** |  | **<477.189** | **≥477.189** |  |
| **Age**  **< 50 y**  **≥ 5 0y** | 9(56.25)  9(47.4%) | 7(43.75)  10(52.6) | 0.853 | 10(62.5)  7(33.3%) | 6(37.5%)  14(66.7) | 0.152 | 7 (43.75)  11(52.4) | 9(56.25%)  10(47.6%) | 0.850 | 10(58.8%)  9(42.9%) | 7(41.2%)  12(57.1) | 0.514 | 11(64.7)  8(38.1%) | 6(35.3%)  13(61.9%) | 0.191 |
| **Gender**  **Male**  **Female** | 11(57.9)  7(38.9%) | 8(42.1%)  11(61.1) | 0.408 | 9(47.4%)  7(38.9%) | 10(52.6)  11(61.1) | 0.850 | 8(10.5%)  11(19.0) | 11(21.1%)  7(23.8%) | 0.408 | 10(50.0%)  9(50.0%) | 10(50.0)  9(50.0%) | 0.745 | 10(52.6)  9(47.4%) | 9(47.4%)  10(52.6%) | 1 |
| **Stage**  **I, II**  **III**  **IV** | 3(50%)  5(33.3%)  8(57.1%) | 3(50%)  10(66.7)  6(42.9%) | 0.425 | 1(14.3%)  7(50.0%)  7(53.8%) | 6(85.7%)  7(50.0%)  6(46.2%) | 0.199 | 2(28.6%)  10(71.4%)  5(35.7%) | 5(71.4%)  4(28.6%)  9(64.3%) | 0.0830 | 3(42.9%)  10(71.4%)  7(46.7%) | 4(57.1%)  4(28.6%)  8(53.3%) | 0.287 | 1(14.3%)  9(64.3%)  8(53.3%) | 6(85.7%)  5(35.7%)  7(46.7%) | 0.0915 |
| **LDH**  **< 400**  **≥ 400** | 7(31.8%)  8(53.3%) | 15(68.2)  7(46.7%) | 0.333 | 7(35.0%)  11(64.7) | 13(65.0)  6(35.3%) | 0.141 | 11(15.0)  10(15.0) | 9(25.0%)  7(20.0%) | 0.921 | 7(36.8%)  11(61.1%) | 12(63.2%)  7(38.9%) | 0.251 | 8(40.0%)  10(55.6) | 12(60.0%)  8(44.4%) | 0.526 |
| **B2M**  **< 4**  **≥ 4** | 3(30.0%)  2(28.6%) | 7(70.0%)  5(71.4%) | 0.633 | 5(50.0%)  5(71.4%) | 5(50.0%)  2(28.6%) | 0.701 | 7(70.0%)  4(57.1%) | 3(30.0%)  3(42.9%) | 0..975 | 6(60.0%)  5(71.4%) | 4(40.0%)  2(28.6%) | 0.975 | 5(50.0%)  5(71.4%) | 5(50.0%)  2(28.6%) | 0.701 |
| **HCV**  **-ve**  **+ve** | 8(44.4%)  6(46.2%) | 10(55.6)  7(53.8%) | 0.786 | 7(38.9%)  8(57.1%) | 11(61.1)  6(42.9%) | 0.503 | 8(42.1%)  9(64.3%) | 11(57.9%)  5(35.7%) | 0.364 | 9(47.4%)  7(50.0%) | 10(52.6%)  7(50.0%) | 0.839 | 9(45.0%)  8(61.5%) | 11(55.0%)  5(38.5%) | 0.567 |
| **Splenomegaly**  **-ve**  **+ve** | 1(12.5%)  17(58.6) | 7(87.5%)  12(41.4) | 0.055 | 3(37.5%)  14(48.3) | 5(62.5%)  15(51.7) | 0.888 | 6(85.7%)  13(43.3) | 1(14.3%)  17(56.7%) | 0. 109 | 5(71.4%)  13(46.4%) | 2(28.6%)  15(53.6) | 0.595 | 4(57.1%)  15(48.4) | 3(42.9%)  16(51.6%) | 1 |
| **Bsymptoms**  **-ve**  **+ve** | 8(38.1%)  10(62.5) | 13(61.9)  6(37.5%) | 0.254 | 8(40.0%)  9(52.9%) | 12(60.0)  8(47.1%) | 0.648 | 12(18.2)  7(5.9%) | 9(27.3%)  9(17.6%) | 0. 634 | 11(50.0%)  9(56.3%) | 11(50.0)  7(43.75) | 0.958 | 12(57.1)  7(41.2%) | 9(42.9%)  10(58.8%) | 0.514 |
| **Reticulin**  **-ve**  **+ve** | 6(42.9%)  7(70.0%) | 8(57.1%)  3(30.0%) | 0.368 | 7(50.0%)  7(70.0%) | 7(50.0%)  3(30.0%) | 0.575 | 9(64.3%)  3(30.0%) | 5(35.7%)  7(70.0%) | 0.214 | 8(53.3%)  5(50.0%) | 7(46.7%)  5(50.0%) | 0.775 | 8(57.1%)  7(63.6%) | 6(42.9%)  4(36.4%) | 0. 934 |
| **BM infiltrat.**  **-ve**  **+ve** | 13(54.2)  5(38.5%) | 11(45.8)  8(61.5%) | 0.570 | 10(41.7)  7(53.8%) | 14(58.3)  6(46.2%) | 0.715 | 11(45.8)  8(61.5%) | 13(54.2%)  5(38.5%) | 0. 570 | 12(50.0%)  8(57.1%) | 12(50.0)  6(42.9%) | 0.929 | 10(43.5)  7(50.0%) | 13(56.5%)  7(50.0%) | 0.963 |

Lymphocyte monocyte ration (LMR) (Lym/mon) , Neutrophil monocyte ration (NMR) (Neut/mon), Platelet lymphocyte ratio (PLR) (Pl/lym) , Neutrophil lymphocyte ration (NLR) (Neu/lym), Systemic immune-inflammation index (SII), Diffuse Large B Cell Lymphoma (DLBCL), micro RNA (miRNAs), Bone Marrow infiltration (B M infiltration), Lactate Dehydrogenase (LDH), Beta 2 Microglobulin (B2M), Hepatitis C Virus (HCV).
