# Supplementary material for: Lymphocyte to monocyte ratio predicts survival and is epigenetically linked to miR-222-3p and miR-26b-5p in diffuse large B cell lymphoma
Source: Sci Rep. 2023 Mar 25;13:4899. doi: 10.1038/s41598-023-31700-x (PMC10039925; doi:10.1038/s41598-023-31700-x)
Supplement: Supplementary file 8 — Supplementary Information 8. [file 41598_2023_31700_MOESM8_ESM.docx]

**Supplementary Table (S8): Relation between LMR, NMR, PLR, NLR, SII and, treatment outcome, tumor recurrence, tumor progression and mortality in the studied DLBCL.**

| **Markers** | **Treatment outcome** | | **P value** | **Tumor recurrence** | | **P value** | **Tumor progression** | | **P value** | **Mortality** | | **P value** |
| --- | --- | --- | --- | --- | --- | --- | --- | --- | --- | --- | --- | --- |
|  | **Sensitive + Partial** | **Resistant** |  | **Recurrent** | **Non-recurrent** |  | **Progressive** | **Non-progressive** |  | **Died** | **Alive** |  |
| **Lym/mon**  **< 3**  **≥ 3** | 12(80%)  8(50%) | 3 (20%)  8(50%) | 0.170 | 4 (26.7%)  2(13.3%) | 11(73.3))  13(86.7%) | 0.648 | 2 (50%)  4 (50%) | 2 (50%)  4 (50%) | 0.540 | 6(31.6%)  1(7.1%) | 13 (68.4%)  13(92.9%) | 0.205 |
| **Neu/mon**  **< 7.4**  **≥ 7.4** | 11(73.3%)  9(56.25%) | 4 (26.7%)  7(43.75) | 0.536 | 4(30.8%)  2(12.5%) | 9(69.2%)  14(87.5%) | 0.455 | 3(60%)  4 (66.7%) | 2 (40%)  2 (33.3%) | 0.688 | 1(7.1%)  5(27.8%) | 13 (92.9%)  13(72.2%) | 0.304 |
| **Pl/lym**  **< 134.273**  **≥134.273** | 9(53.0%)  10(71.4%) | 8 (47.0%)  4(28.6%) | 0.495 | 3(20%)  3(21.4%) | 12(80%) 11(78.6%) | 0.716 | 4 (50.0%)  2 (66.7%) | 4 (50.0%)  1 (33.3%) | 0.852 | 1(5.9%)  5(33.3%) | 16(94.1%)  10 (66.7%) | 0.406 |
| **Neu/lym**  **<2.131 ≥ 2.131** | 9(56.25%)  12(70.6%) | 7(43.75)  5(29.4%) | 0.621 | 3(20%) 2(13.3%) | 12 (80%)  13(86.7%) | 1 | 5 (62.5%)  3 (75%) | 3 (37.5%)  1 (25%) | 0.828 | 1(0%)  6(33.3%) | 15 (100%)  12(66.7%) | 0.127 |
| **SII**  **<477.189**  **≥477.189** | 11(64.7.1)  10(66.7%) | 6(35.3%)  5(33.3%) | 0.797 | 4(28.6%)  2 (11.8%) | 10(71.4%)  15(88.2%) | 0.470 | 2 (28.6%)  4 (80%) | 5 (71.4%)  1 (20%) | 0.156 | 1(6.25%)  5(29.4%) | 15(93.75%)  12 (70.6%) | 0.203 |

Lymphocyte monocyte ration (LMR) (Lym/mon) , Neutrophil monocyte ration (NMR) (Neut/mon), Platelet lymphocyte ratio (PLR) (Pl/lym) , Neutrophil lymphocyte ration (NLR) (Neu/lym), Systemic immune-inflammation index (SII), Diffuse Large B Cell Lymphoma (DLBCL) Diffuse Large B Cell Lymphoma (DLBCL), Number (n).
