# Supplementary material for: Lymphocyte to monocyte ratio predicts survival and is epigenetically linked to miR-222-3p and miR-26b-5p in diffuse large B cell lymphoma
Source: Sci Rep. 2023 Mar 25;13:4899. doi: 10.1038/s41598-023-31700-x (PMC10039925; doi:10.1038/s41598-023-31700-x)
Supplement: Supplementary file 9 — Supplementary Information 9. [file 41598_2023_31700_MOESM9_ESM.docx]

**Supplementary table (S9): Common and unique target genes for the 3 studied miRNAs miR-222-3p, miR-26b-5p and ebv-miR-BHRF1-2-5p.**

| **miRNA** | **Target genes** | **Total number** |
| --- | --- | --- |
| **ebv-miR-BHRF1-2-5p, miR-222-3p miR-26b-5p** | CORO1C MYC PANK3 YOD1 DICER1 ZNF451 TET2 CDK5R1 ETNK1. | 9 |
| **miR-222-3p**  **miR-26b-5p** | BTG2 MMGT1 G3BP1 GALNT3 MESDC2 CHORDC1 TRIM33 OSBPL8 TMEM19 EIF4G3 CAND1 KPNA2 TSPAN13 TPCN1 BACE2 UBN2 HSPA8 ZNF711 YWHAH TNKS2 TMEM248 HNRNPA0 NSD1 TLK1 BAG3 PSMD12 IPO5 KIAA0368 FBXO3 HIPK3 MME ZFAND6 VCP STMN1 WEE1 CCDC47 HNRNPH1 NUCKS1 YWHAG SFXN1 FNIP2 ETV3 HNRNPA2B1 BIRC6 MDM2 MAML1 DDX3Y CPSF6 PPP2R2A FAM134C TMBIM6 C16orf72 SEMA6D ZNF616 ERLIN1 RNF24 METAP2 TFAP2A SLC25A36 CSNK1G1 RC3H2 PPP6C BRD2 ADAM17 FAT1 PTPRJ FNDC3B CAV2 MIA3 SFT2D2 GSE1 PMP22 HOXB7 LPCAT1 CYR61 UBR4 CTTN KMT2C SATB1 BRWD1 EIF4EBP2 QARS RGMB ANXA1 RASSF3 ALPK3 CHSY1 LNPEP MINK1 SGPL1 TIPARP MIER3 RAI14 CDH2 NACC2 TCF12 NUPL1 CSTF2T ZFP36L2 HUWE1 CDK6 SMARCA5 SNRPD1 REV3L SCD SGTB FAM168B OSTM1 ZSWIM6 SLC4A7 RQCD1 TXLNA ATL3 FAF2 RPRD2 KIF3B UBE2D1 CREBL2 CASP3 SEC23A PPP1R2 ASXL3 CBFB SESN3 DDIT4 BMPR2 SLC16A6 ZBTB33 FOS TP53INP1 DDX6 TROVE2 ZNF460 SLC20A1 ZNF367 DST HOXA9 CDKN1C SRSF2 ZC3H11A MTERFD2 DYRK1A CDV3 BTBD7 SLC6A9 POFUT1 ZFHX3 GMNN SERINC1 SIX1 HDGF RNF19A FICD IGFBP4 UBE3C PHF10 LARP4B RFX7 TRPS1 SERBP1 DDX3X E2F3 HIPK1 WASF2 TOP1 SLC39A6 | 166 |
| **ebv-miR-BHRF1-2-5p, miR-222-3p** | TRAM1 UBE2J1 YES1 CLIC4 PTBP3 ZNF121 C18orf32 FNDC3A QSER1 STRN BIRC3. | 11 |
| **ebv-miR-BHRF1-2-5p, miR-26b-5p** | SLK SCP2 B2M COL19A1 GNA13 FKTN ELK4 LMNB1 G3BP2 TNRC6A OAS2 PDE7A PMAIP1 SUCO CDKN1A SFMBT1 AKAP11 RCN2 VGLL4 SOS1 RAC1 DUSP22 LRRC58 IPMK MTMR9 IRF4 PRR5L MGAT4A INPP5D BACH1 VANGL1 FAM217B MEX3C ANO6. | 34 |
| **miR-222-3p** | RPS17 CEP250 BRIX1 NHLRC3 FAM214A SCAF8 DAZAP1 ZNF131 DIRAS1 COX15 ARL6IP1 LHFPL2 ZNF510 HOOK3 RELT RBM17 FAM73A METTL2B CEP170 HECTD2 FRS2 RNF4 EIF1 CDK1 HYOU1 SF3B6 TMEM59 RCSD1 BRK1 RAB5B SDC2 QKI GNG12 NUP88 ZCCHC9 ZNF227 PRRG1 SUV420H1 GNB4 NKTR PHF1 FAM126B SERTAD2 POLD2 TNRC18 CEBPD C12orf65 GANAB TMED7 PDGFD RARS2 KIT SPAG9 SLC39A9 GNS CTNNB1 ELF1 FOXN2 VPS16 EAF1 TRIM35 NOTCH2 PSIP1 TEX10 FAM199X CUL3 NFATC3 SPATS2L CDS2 SUPT16H NUF2 WDR82 PKP2 SLAMF6 C9orf156 STK39 MYBL1 CDC27 PPP1R15A KPNA4 BMF GLO1 FLT1 PFAS KMT2D C6orf89 USP15 GTF2H1 HECTD1 ERCC4 TXLNG TLE3 RCHY1 TACC1 SERTAD4 CCNJ DHX8 SEC24B MEX3A EEF1D UNC5C ZFP36L1 TIAM2 C10orf2 HSPA14 IQCJ-SCHIP1 ATF2 DEGS1 THBS1 RBBP7 SLC30A1 SP2 GTF2B ZBTB10 LRSAM1 GGCT SLC35D1 MAP3K4 CLCN3 SETD1B XRN1 RBFOX2 POLR2A ZFYVE16 ADIPOR1 TAF3 ABHD10 VCAN TMEM64 MKI67 BICD2 ENTPD1 PPP4R2 p27(hsa) TAB3 C20orf166 FAM53B PTPRM ATXN1 TPM2 CELSR2 CIAPIN1 STAMBP NFYB RAB1A BMI1 CD164 EIF3E HMGCS1 KDELR2 EIF1AD p27kip1(hsa) DEK DCN ZNF91 LIN7C MORF4L1 ZC3H15 MAGED1 TNPO2 HNRNPU CSNK2A1 TMOD3 DDX20 OTOG USP8 ARID1A CYP1B1 C1orf43 RBMS1 AGO3 PGBD4 HDLBP OAT UGCG IRS4 FAM178A MACF1 MTA2 AP1G1 KCNAB3 CD4 DPYSL2 SERINC3 NRP1 GJA1 GNAI2 CLDN11 TIMP3 SQSTM1 PAIP2 TMEM127 SLC35F6 NUP93 MRPL19 RAPH1 ZNF749 MFAP3 ARNT MARK1 CDKN1B FOXC1 RBM4 COPA SLC40A1 ARF4 CCSER1 NKRF COPZ1 WDR75 TSC22D3 RAB11FIP2 ETS2 ARF1 ATP7A ORMDL3 ITGA8 RASEF PABPC3 SSR3 ZCCHC24 IFIT2 MTF1 NEK7 ASPH FAM222B NR2C2AP LEPROT ADAMTS19 VAPB PRKAB2 ZNF76 STC1 PLEKHA3 SNX4 CLDND1 PPP2CB ZRANB1 LDHA PHC2 UBA3 EIF5A2 SLC44A1 CNNM4 PRPS1 OXR1 MIDN FAM53C TUB DCAF7 AGFG2 NAPEPLD UBXN2B PER2 DKC1 NFIC SUN2 BCOR ATF4 SEH1L CTCF HEG1 FUT10 PIN4 TAF6L ZNF629 HMGB2 TFG KARS UBE4A ZCCHC14 ICK FERMT2 TPD52 STX3 PHACTR4 PRKAA2 VEZF1 MYLIP FAM35A BCL2L11 LYN SDCBP HNRNPF RREB1 LIFR SNAP23 DCUN1D1 MBNL1 SDC1 FAM46A ZNF226 HSPA5 SECISBP2L PFDN6 ESF1 AP3M1 ANAPC16 ICAM1 ALDH1A1 ABHD3 SCARB2 APLP2 NCAPD2 PAFAH1B2 ETS1 CDKN2AIP MOB1A EPM2A CD81 FRK TRIM71 TMED7-TICAM2 H1FX TRIM32 ZEB2 XBP1 BUB1B C1orf35 RRP12 DPP8 CMBL ANKRD12 UBTD2 ACVR2B IRGQ FAM83D RNF219 PRELID1 MLEC RPL41 HTRA2 WSB2 JAK3 ZFR CANX P2RX4 PABPC1 LYSMD1 CCT5 SNRPB ULK3 TIMP2 SEP15 ZNF330 SPARC KCTD12 HES4 ZBTB5 B3GALNT2 PSME3 NUP43 RPS14 ANP32B SESN2 TAOK1 RNF44 UBE2J2 ZNF638. | 359 |
| **miR-26b-5p** | C9orf152 FSTL1 FARP1 VPS33A TUBD1 POLR2G GNL3 RXFP3 APBB2 XDH CNBP RTKN2 MTERF PFKP COX7A2L PMPCA C9orf91 ANKRD36B PDCL3 RNF38 GFOD2 AEN CXCR4 SALL1 CRTAM HSD17B2 LMAN1 UBE2G1 PAK1 MSL1 PELI2 SIDT1 TPX2 EP300 CCNI NTSR1 NKX2-5 GYS1 CENPQ OR2W1 TRIB3 TAF9B DDX5 DHX35 COL4A5 DEC1 CCNB1 UBB CDK14 TMX4 SOD2 CYTH1 CHD9 C1orf27 STK16 XK MREG CEACAM6 CERK SPECC1L TET3 PCSK1 RAB40B TNS3 IGSF3 PIKFYVE LONP1 ARF6 YEATS4 CXCL13 OAZ2 PTCD3 NFIA HMGN1 CHD1 C10orf12 CXADR SUGCT ECH1 EPT1 DHRS11 PCDHAC1 ARL4A BLOC1S2 ACTR8 DAD1 CYP2F1 ITGA3 RABGGTA KIAA1033 CDC25B CYP2D6 MAP1LC3C CRYGC GINS1 C5AR1 ACSL3 UTRN TRIQK BAZ1B ABCD4 ROCK2 SNX16 ANXA3 TLR1 PPP1R15B ZIC2 CLTA EED HES1 FGGY CA11 SIX5 ACBD3 TMCO3 DSCC1 HSPA1L CIAO1 KIAA0020 DDB2 FITM2 STARD5 MKNK1 SLFN12 FAM98A PCDHA13 FOXE1 RIPK4 ZDHHC6 CCNE1 ALX1 NPC2 EVI2A IL17RC RBPJ LRRC8D FOXM1 BUD31 AP3M2 USP14 TPI1 RASGRF1 ZNF556 FBXO11 CDK8 C6orf15 FIGF EMP2 C3 QDPR PPP1R3D TPM3 SLC6A15 POLE3 HSPD1 CHST15 SLC38A10 PRY C1orf216 SIPA1 EP400 PTK2 RNF217 GDF10 GPR75-ASB3 COQ9 NELFE UBR3 LAMP3 DESI1 CDIPT OTUD4 PMF1 FAM98B ZSCAN16 CD82 COPS4 CBLL1 BCHE ENOSF1 EIF1AX PPM1A CPEB3 SRSF11 PCDH7 RPLP1 MMP16 CAMTA1 ADI1 TMEM156 SLC35D2 DRD3 SON KIAA1324 ALCAM ABHD2 QPRT MZT1 IL24 C4orf29 ZNF605 WDFY2 SLC6A6 RACGAP1 CHFR ID1 GKN1 CDS1 RAB3A COX8A ZNF35 C9orf16 ERBB2IP ADAM19 FKRP SSX7 PRPS2 FBXO24 THRB DNMT3B MYBPC1 TSPYL4 DNAL4 CELF1 ADRA2A TASP1 SLC13A4 PPP2R5A POLG ZNHIT2 POTEG SPTBN1 HSF2 SRP72 ZNF24 MTX1 PIP4K2B BAMBI PRRC2B GRIK2 CYP2D7P1 LTBP1 TRIP12 PCDH18 GNRHR SLAMF1 RASIP1 DLGAP4 ZBED1 PHLDA1 LIF CCDC181 CNTFR PSMC3IP H3F3B NUDT12 ENDOG EZR ZNF549 ZBTB18 OFD1 FGFR3 AGT CLSTN2 ANXA8L2 CST3 CDC6 C12orf4 UFL1 KDELC1 COX10 DNAJA3 GLG1 KIF2A HPS4 INPP5J EPHA4 FANCC ARL6IP6 NEBL GPATCH2 MRPL15 CNOT6 ZBTB20 PNOC KRT222 MED14 HP1BP3 PCCB BRD8 SPRY2 WDR12 PJA2 HOXD13 GSTP1 GMFB CEP85 CHMP7 DCSTAMP SF3A3 NOC3L PBLD STON1 AHNAK AAMDC ACTR5 TENC1 FN1 PSMD5 C17orf53 LACTB2 CBX6 EIF4A3 CAMSAP1 RTCB ATP1A1 ZNF672 NFE2L2 NEK6 SETD8 YWHAZ CYP4A22 ANKRD52 PAQR4 ACSF2 KLRC1 JADE1 RPS6KA6 SH3D19 IFI16 GOT2 GALR2 PRPF39 GABBR1 ATP7B LSM3 GPBP1L1 CARD14 FSTL5 CHMP6 SNAI1 CCDC6 BRD4 SPCS3 EFNA2 ZDHHC11B ARFRP1 CHAF1A ENTPD7 ZFPM2 DEFB4A SEC63 MRPS28 FTH1 DTNB NPRL2 GRWD1 ITSN1 TMEM170A DNAJC11 PCBP3 ASAP1 FAM114A2 LRRC2 WDR91 HAGH NCAPD3 RGS20 TRAM2 AGO2 MTMR12 KLHL21 ASTN2 PARK7 DUSP14 ELAVL2 RPN1 PSMC4 KCNH4 FAM160B1 EIF3D DDI2 MPC2 NOP10 KIF4A POLR3G SNRPC CPD ANXA8 DEFA1B AOC1 FAM193A DNAJB4 NTN4 CYB5R4 TRAPPC2 ZNF518B SERP1 KRT8 LMAN2L C1GALT1C1 MAP7 EIF2S1 POTEJ GPR135 LGR5 TAF9 TERF2 DGAT1 TAC3 LRP1B BARD1 PHGDH IGFLR1 XPO4 EPS15 CA9 TLX1 WNT5A THOC7 TCTA KIAA0319L FDFT1 PTGS2 IMPAD1 UBE2D3 TEX2 KIAA1551 AC008132.13 NELFCD MAP4K3 HOXA5 GALNT2 ATP6V1A INPP5B GABRE IL20RA ELF4 ANO10 CCNK AL590762.11 PARP4 CD200 MARCH3 ICE1 ZNF468 C1GALT1 OXA1L THAP5 CCND1 SLC9A2 ZNF136 USP18 SLC30A7 RASSF9 NET1 BMP2 TOX MVD MGP CCDC144A GCNT2 GABRG3 SLCO1B1 CCNDBP1 KIAA0232 SMS ERCC8 RRAGC GPX4 ACE2 GCH1 CHMP3 ICT1 TYMS VPRBP ALG9 ANAPC1 RPL39 CPA4 RBM3 GPR1 PODXL SCFD1 TRPC4 ZNF551 GRTP1 PCDHA12 NR1D2 KLRC4 BSDC1 POFUT2 PRDM13 METTL16 DSC1 FRS3 MAP2K4 PTPN4 ITGB2 FOLR2 TRANK1 UBE2K SF3B2 XPOT GAL3ST1 ZNF480 PSMB8 ZWINT TRIM14 TBCCD1 OR10H1 ENPP2 SLC7A11 DERL2 OR10H2 FHL5 UXT DCUN1D2 SLC6A16 LIPT1 LRRC8E MAST4 MPDU1 COG8 SLC5A3 TRMT2B CHST2 ZBED6 ZNF18 WDR26 NAA15 NR4A3 BLOC1S5 MCTP2 BCL11B SOCS3 ZNF839 POP4 MEP1A ACAA2 IL10RB PARK2 PCDHA7 TMEM132A TIMP1 PLOD2 TRAPPC6A CEMP1 E2F7 LAMA3 SMG1 SCGB1D2 SP4 PIP5K1C KIF1B GSK3B VPS37B ABHD14B TNFAIP3 NAA40 KIF5C PDK4 MAPK1IP1L IBSP FUT8 TRMT11 CCDC28A BEND4 TESK1 SNURF CEP104 GCLC CNOT2 TMEM62 POLR2M IKBKAP POLD4 COLEC12 LIX1L BLVRB GALNT10 LSAMP CWC25 METAP1 TRIM6 APOF SSFA2 TP53I3 SMURF2 GREB1L ARHGEF5 MRPL22 ADAMTS12 ZNF862 BBX AVL9 TULP2 TNRC6B FOXG1 BNC1 TRIM24 COMMD8 PLXNA4 NAP1L1 ECT2 SLC38A6 SEPT7 ASPN CCT7 ZFX TRIP6 CDK18 KDM4A URB2 EI24 ZNF548 GREM2 KIAA1045 MTF2 HOXB1 CRADD INPP5K DUSP12 CASQ1 BLNK CACNA1C FIG4 FAM20B IGHMBP2 FOXD2 C6orf211 GGH INTS7 CCSER2 BRI3BP ZNF664 GPC4 AP1S3 LMO7 ASNA1 COL4A2 ADRBK1 SLC1A6 ZNF84 RBM4B HMOX1 SLC27A6 RBMY1A1 ERAP2 DYRK3 INO80B ATP11B NLGN4X ADGB RCOR1 MKNK2 ZNF44 RMND5A HEATR5A IMPG2 BGLAP ZIC5 SLC35F5 CCR6 TSPAN12 NRN1 CNTLN METTL14 TFAM SRP14 ACADM CDC14B SLC22A23 KDM7A FAM8A1 CXCR1 CEND1 ZDHHC7 VKORC1L1 FAHD2B MMP8 ADAM29 CTNS CBX4 SCAND2 MYO9A PFKFB2 MIER2 KIAA1644 CES3 LIAS CNPY3 PLCB4 MRM1 ATP9A TSKU PDLIM1 GADD45A DPM1 PSMB7 IAPP TSKS PPP1R14B NHLRC2 GP1BB TAS2R9 ZNF793 TRIM6-TRIM34 POLR1B GTF2A2 RHOU CYP27B1 CALCB VPREB1 PPP3R1 EPC2 DMXL2 SPRY1 KRT4 NAE1 POTEF SLC17A6 FMO3 POU4F3 COA4 ITGBL1 IPPK SRP9P1 KLHL15 GALR3 ABCA6 SESN1 TMED8 APPL2 TNPO1 ENOX1 CMC2 ZNF706 SNX5 NCAM2 AFAP1 C12orf5 CHML PGAP1 MSRB2 PAPD7 CAPN2 TWISTNB CHD2 MTMR4 SLC25A17 FZD5 POLR2F EMC10 BNIP1 TRIM17 FAM134A KIAA0391 PURA KRTAP5-8 DNPEP CELA3B GTF2IRD2B SAMD11 HOXC4 TSC22D2 UBQLN3 SMAD1 RMDN1 NDUFV1 MYT1L CFL2 UFSP2 STAG1 NAP1L5 DCBLD1 SORCS3 SLC2A6 SKIV2L2 HIST2H4B ABCF1 CD28 DIABLO BAZ2B SOHLH2 TAGLN3 FAHD2A BRD7 AGL ZDHHC20 GBP1 DEFA1 CEP55 KIAA1377 TAX1BP3 F13B CCNB1IP1 EYA3 TNP2 EML4 HIST1H2BI OR10H5 NUDT19 SLC22A13 SKI PLEKHB2 TUSC3 RPL28 SEC61G NOX3 TMEM50B TBL1XR1 HELB G6PD LINC00483 DDIAS NUDC SCARA3 DENND6A EMP3 SYNCRIP PVALB ELOVL2 MED13 APOO CAMSAP2 MTAP RP11-663P9.2 ACSL1 TRNT1 SNAPC5 TXNRD1 TNFRSF21 SRGAP1 SLC16A4 BRDT SLC6A8 SMPD3 DNAJC21 TAZ CRIP2 NT5DC2 AP000346.2 ZNF423 ELMO1 PPP1CB LRP2 CYTIP MTDH MAP3K12 UNC13A CHST7 SLC3A2 TARBP2 NDUFA1 TRIOBP IER5 SYNE1 ZNF573 S100A2 RBM5 TUBGCP4 TSPYL1 ERO1LB MOBP GUF1 GSPT1 GUSB DEFB4B KRTAP5-3 GSDMB LIG1 CRELD1 SUZ12 GDF11 MRPS16 KRT18 KIAA0922 TMEM74B HTRA1 BHLHE40 ZNF585A MCC RAB32 RNF111 P2RX2 DDX43 CAMK4 KANSL3 HHLA3 CHST12 TARDBP NME5 ATXN1L RB1 B3GALT6 RP11-624L12.1 PHLDA2 CSPG5 CNIH4 ADAR BAHD1 MPV17 GAS2 CACNB2 NMRK2 GLRX SHOC2 PDHX NTNG1 ZBED4 ITGAX NR0B2 NABP1 MTPN CANT1 CDK2 IRF2 SLAMF7 PGR ADAM18 ZNF507 TMED10 SRPX LRRC8B LRP12 KIF13A SDC3 RPA3 YIF1A MICU2 MSMO1 RDH14 PTPLB SYNJ2BP RAD51AP1 ZSCAN31 DDX17 PDE4DIP EMC6 LRRC1 PKIA IRGC CATSPERB GABRB3 TIPRL MED31 GALT P4HA1 GPR17 LRRC20 ATP6AP2 LPPR1 SLC35C1 IL36RN RSPRY1 RNASE1 NDUFB11 UBE2A VPS54 ADAP2 TM9SF3 TAF8 KANK2 GLI2 RBM41 SCO2 ZFAND5 BCL7B EIF1AY MAFG SPAG1 KIAA1024 OR51E2 TAS2R13 SLC41A1 PRRG3 GDE1 RAPGEF5 DAK PLGRKT PURB CHIC1 KBTBD2 BAG5 RELB SDE2 SLC39A10 DTD2 PDIA6 PBXIP1 MCUR1 CASP9 SRPR EID2 BAG4 KCNN2 DYNC2LI1 SLC2A4RG DGKE MRPS15 EIF2B1 CPOX TBC1D15 FAM214B MAGEA11 LYZ SH3BP1 MYOG ZC3H4 KLHL42 NCOA2 S100PBP GATA2 TOR4A AJUBA EBNA1BP2 PDGFRA HELZ AGPAT5 TET1 PTRH2 ADM CTDNEP1 TMEM50A ABCB6 CRYL1 PPIC OTC IMPDH1 PRKACA RORC FZD3 SOCS5 IFIH1 IDH1 NUAK2 DLG5 MANSC1 TFEB IMP3 INHA SMARCAD1 CNIH1 SH3RF1 MTCP1 VMA21 LMCD1 GPR63 ZHX1 NFKB1 AREL1 ARHGAP35 TMEM167B FAM46C ZKSCAN3 NEK9 S100A7 WDR83OS TDRD7 HARS2 TPRKB GNB1 UPF3B CXCR6 FKBP9L PIDD1 IGSF6 TPPP IGF2R ADNP UST VIPAS39 RAMP2-AS1 AFTPH LCAT ERICH1 ADPGK VWA9 LARP4 NSMAF SKIL TGFB1I1 KIAA1462 PYGO1 TLE4 DDX25 TOP2B TMEM192 SH3GL2 RAD54L2 IGFBP3 TOLLIP TRAPPC3 LY6D C16orf70 KIF22 TMEM132D RNF11 CERS6 SEC61A2 HIST1H1D FOXF2 EPAS1 RAB18 VAMP7 USP32P1 PLA2G12A CYB5A PITPNC1 RSF1 KIAA1598 POU2F1 HSPB7 NKX2-2 CNNM2 DPPA4 C14orf2 IFRD2 CBLC RRP15 PTCHD1 GALNT1 SLC39A1 NARS2 LIN28A FAM127B CASP4 SEC24A KIAA0101 TSFM WRB TSPAN17 ZBTB25 MTX2 VNN2 MCMBP FBXO28 BRD3 BNC2 WDR3 CPN2 PCOLCE2 NAT1 WFDC6 TRAFD1 CYBRD1 SPTSSB MTCH2 MMP10 LBH ZFY SYNM LPHN2 HSD17B14 UGT8 STON1-GTF2A1L BMP2K SLC25A23 C8orf33 HEXA PHLDB2 NRD1 TMEM56 ATXN2L CHPF EPM2AIP1 RBM28 KRTAP5-7 SRRM2 MRPS18B AAK1 AOX1 COL1A2 TMEM208 EEA1 TRIM36 GIGYF1 CAAP1 TMC7 KTN1 MYBBP1A MAB21L1 HSBP1 ARL5A PHF11 RUFY2 SF3B3 RBAK NDEL1 RANBP9 PIM2 GJA5 SLC17A5 HGF C4orf46 ZNF503 FKBP2 SI ARSJ KCNE4 ACSL4 RBM48 HSPA12A REPIN1 ARL8B STARD7 ERC1 SMIM13 NKIRAS2 YPEL3 PPM1D NDUFA5 CH25H KAT2B COL12A1 TMEM206 SMAD6 CHST3 BTAF1 ROS1 RCBTB2 RBMY1HP BMP8B C1QTNF1 UQCRC2 RYBP STT3B SLC19A2 PSD3 CPSF2 B3GNT2 LTA4H SAFB GNB3 ZNF224 TRMT2A ZNF652 TRIM68 MEF2C RNASEH1 PGK1 WLS RLF MLXIP KCTD14 XRCC2 KHNYN MTFR1 C11orf16 B3GNT5 ANKS1A SMNDC1 DONSON LTV1 RYR3 BCL2L12 ACTR3 CCDC25 OTUD1 HECA RAE1 DFFB NDUFB1 MEF2D TLX3 BID OR2S2 RCBTB1 COX5A FGL2 EIF6 AIFM1 FAN1 DCHS2 GSTA1 RP11-168J19.2.1 TSTA3 LILRA4 TOMM20 MNAT1 NUFIP2 PTEN TAF4B ATP6V0D1 EEF1E1-BLOC1S5 SUSD5 AGFG1 GTF2A1 KIAA1279 ATP1A3 MEX3B PNKP NUBP2 ROGDI S100P MATR3 CA13 ZNF85 SERPINB5 GPR107 PLCB2 TIA1 TXNDC15 RECQL HLTF ENPP4 KCNF1 KLF11 SYNE2 GPR180 C20orf24 GALK1 GABARAP TPD52L2 IRF1 FBXL6 GOLM1 MAGEC3 SSH2 SDHB C5orf24 TSPAN3 CYLC2 FLVCR1 USP20 DUSP7 DERA TRIM22 RNASE6 ATAD2B TAS2R10 PHLPP2 LAPTM4A GPNMB SFPQ RAB3GAP1 EXT2 DIAPH2 PTPN12 ABL2 CLN8 ASCC3 PSMA1 ALKBH1 GRM8 SLC23A2 RNF141 CSN3 PKD2 TMEM86A EPHA2 RGS3 ZNF277 ARHGEF12 SEPT10 NMRK1 SNRNP70 PRICKLE3 RPGRIP1 CSGALNACT2 KCND1 CMIP PHKA2 CTDSPL2 ATF3 RSL24D1 YIPF4 THAP10 CDK12 MRPS34 ANKRD10 FBN2 PHF3 MDH1 DCBLD2 RBM33 BTG1 YTHDF3 KLHL23 ZNF148 CNOT4 ZNF141 ZBTB38 TMEM30B PGRMC2 IKZF1 PAQR6 GGA3 OSTF1 PCDHA4 HIST2H4A FOLH1 MAPKAP1 THOC2 SLC16A12 COL15A1 GLRX2 TRPV6 EXOC6B GAN PCMT1 GRB7 HDAC5 YWHAE ATP1B1 ARMC1 GREB1 PM20D2 REEP3 C17orf59 MGST3 PNMT NDUFS8 ABCA1 PAK6 ACBD5 PQBP1 CLEC2B REST FNIP1 EXD2 PPAP2A PRY2 RTN3 ULK1 PRYP4 TEAD3 SLC2A3 PCDHB8 ATP13A3 CD36 EFNA4 KIF5B RBMY1F MRPS17 MUC7 NUP153 DCAF12 ANAPC13 LRRC17 TMPRSS11E TNFSF15 HSD17B11 SH3GLB1 SECISBP2 IDH2 ZNHIT1 PCGF6 SUPT5H COPZ2 URI1 PIK3C3 MYEF2 MEF2A NOP2 ASF1B TAF2 AGPAT3 ANKRD28 MAGI3 CDK2AP2 TRAF3 AC068538.2 LETM1 USP10 SND1 B4GALT1 DHX33 LRFN3 ZSCAN26 FKBP14 HIST1H2BC HOXB3 CTSV GDI1 ZNF81 CASP8 TAP1 CMC4 CYP11B2 BNIP2 DHX30 POTEE FAM135A GTF3A METRN TSGA10 TMCC2 ZNF721 POU4F1 ERAP1 PLCH2 GPR22 NCEH1 FBRSL1 ARMC8 DSG2 ISOC2 AEBP2 HSPA4 PAPPA RNF144A CYP4F8 EMC3 DCTN3 POM121L1P CAPN9 CCL7 PRKCD TRPC7 NDUFB4 RBMS2 MT1M TRIM65 TMPRSS6 MYO10 GPALPP1 SUN1 RBM19 PRKAA1 CASD1 FADS2 HSPA4L IL1RL1 SMC4 PLXNC1 FCGR1B TMEM230 RNF103 NRAS MAPK9 ZNF138 FAM208B KHK FBXW2 NYX PARP14 TOM1L2 ZNF410 TRIM37 GRHL2 TMEM165 MAGEA9 MNDA RAB21 IREB2 PSMC6 KLRC2 PBRM1 CPB2 UHMK1 TADA3 MPC1 HECTD4 MAGT1 PCDHA11 PIM1 BATF3 EPPIN NR2E1 ZBTB43 USP9X DYRK2 RNF2 PIK3R3 PCNX KLF6 USPL1 PIK3C2A FCN2 CCL27 BHLHE22 POLA1 PFN2 PPP1CC FLOT2 SLBP RRAS2 CCDC109B EARS2 OXCT1 SH3BGRL ARPC5 SV2B MCM3 CDKN2D UBR5 PRPF4B NRBP1 STUB1 ZNF679 HIST3H2A CCDC170 SLC22A8 EYA1 IL13RA2 SERPINB2 FES MAPK14 ZNF724P CCNL2 RORB FAF1 ZFHX4 TFPT EIF2S3 SLC25A13 ODAM GPR126 GSTO2 TMTC3 LUC7L TBC1D2B SUB1 AKAP10 MAGEA9B EFCAB14 KIR3DX1 ZBTB45 ST8SIA1 ANKRD46 CPSF3 RSRP1 PLS1 CD248 DGKZ PDE8A BLM RAB23 CHST4 TIMM10B ARPP19 COPS2 SMAD7 SOX3 BOLA2 LARP1 PSMB2 PDGFB KIAA1107 VIM WARS MPI ANO2 MEPCE NCAPG2 NAA50 MAGIX GDAP1 HOXA10 CARS2 PCNA ABCE1 EXOC8 DDX60 SUPT3H HLA-A HSD3B1 PEX13 RWDD3 ATP6AP1 ETV4 KYNU STAT3 SLC38A2 PPP2R5C CD2AP ROBO1 GID4 SMN2 MYH10 SPATA6 RUVBL1 ADAM9 STX11 DDX52 THOC1 FAM212B DNAJA1 HSF4 MATN3 POMK IFNGR2 USP25 DENND4A ANKRD11 ARMC7 TBC1D20 PCYOX1 CEP83 NSL1 DUSP1 H3F3C BABAM1 CYTH2 NPR3 RAD51C CPM CYB561D2 ZC3HAV1L MEGF8 ADAM10 PARP6 NDUFB5 SLC26A4 FAM220A RABGAP1 FBXO5 N6AMT1 GPRC5A RNF187 GBP2 ZNF217 RGCC WDR4 FRAT2 VTN UACA HEXB CD97 SETD5 CRIM1 PTER RRS1 MPHOSPH8 TNKS SMPDL3A SLC35G1 SNX2 CRLF1 CTH AC017104.2 MADD PRRC2C PLEKHA1 KLF12 ARRB1 KCNK1 ZCCHC3 HNRNPUL1 PFKFB3 LIG4 PCDHAC2 SFRP4 IL17RB TMED5 AGTR1 GSTT2 WDR60 TXNL1 RBMY1J AMDHD2 C14orf166 GALNT7 ABCG4 ANKRD36BP2 DENND5B PNRC1 MDM1 MAT2B SRGN VDAC1 PPP1R10 TRIB1 CASP7 LENG8 TXNDC5 TOM1 CD46 RBM12B FAM118A STYX PRUNE2 PMFBP1 SMAD4 WRAP73 ERI2 CTSD STAT6 DNAJC12 ZNF195 CREBZF PER3 IL3 CDKL2 AMACR S100A1 TFRC RPL6 TINF2 EPC1 AK2 VPS13A SPRR1A CYP4A11 HIAT1 RAB36 PFDN5 CMTM6 PPT1 GRB14 MLLT3 UBE2B CCNT2 KEAP1 FBN1 ZNF621 JOSD1 ZBTB39 SNX14 FANCF INHBB PRR4 ARFIP2 SRP19 RPS6KB2 TRAPPC10 TMEM187 DCAF16 SLC6A14 FAM92A1 DIS3 THAP3 SPATS2 LASP1 TCAIM SLC28A1 EGR3 TCEB3B SPIN1 IFRD1 IL12A RAPGEF4 PRR14 KCTD9 EMC7 ZNF696 ZBTB6 MCL1 CCL2 FBXL19 KPNA1 PSMC1 TMEM41B COASY LAMC1 CLTC SRP9 ATP5EP2 DFNA5 PDCD10 EIF2AK3 SMYD2 HES3 ACADS PBX3 CXorf38 ALKBH4 PSME4 ANXA5 BATF MED23 TRIM58 UFC1 PSME1 UGT2A1 CHAC1 TOR1B C9orf114 HMGA2 ETF1 EIF4G1 ING2 SUV420H2 PSTPIP2 PMF1-BGLAP C11orf30 NUP210 SEL1L TRAPPC13 RNF213 PDIA5 CNEP1R1 SLC7A2 PRPF40A DNAJC6 PODNL1 CLOCK LGALSL DEFA3 KIFC3 L1CAM NXPH4 TGM1 TAGLN CPA1 AP5Z1 TK1 PRKCSH FASLG ZDHHC18 ZNF140 UHRF1BP1 IVNS1ABP C1QTNF9B-AS1 BTBD3 FOSL2 ABCG2 MRPL18 PACRG CAMKMT KREMEN2 LSM11 GRB10 SREK1IP1 PDGFRL PCDHA10 CLSTN1 EEF2 PHIP PRDX3 DPF3 HMGN5 ODF2 RFWD3 RBM12 HSPA13 C19orf66 RMI1 AL359195.1 PTPN1 DOCK9 IFITM3 SOCS7 DAB2 SACS ASPM SERPINH1 ZYG11B PLXNA2 DVL3 NIFK ATP5SL MN1 PIAS2 ATP6V1B2 TBCC NSF TMSB15A TBC1D13 LRRTM4 DNAJA2 CACNB4 ZNF597 SCGB1D1 OR2J3 CHM CRYAA BACE1 STX1A ACADSB FAM105A HERPUD1 USP6 POMP HGD INHBA CDCA3 TFB2M METTL9 UGDH FAM198B WBP5 LGMN TCN1 GPR183 PLCB1 NPEPPS TXK NUCB2 TAF13 SOX5 RAP1B ADAMTS1 SFT2D3 P2RY6 DGKI FAM136A GPR27 CFI CDH9 SLC7A5 EIF5 UBL3 TIMM10 NFIB PIK3CG AK4 NLN PIGA ZNF23 COL4A4 KHDC1L TBC1D30 TRIM13 GSPT2 STIL MB DNMBP SP1 GSTT2B GTF2A1L HSPA9 ANXA8L1 WDR25 GTPBP4 DUSP21 EIF4A1 MXI1 GINS4 NDST2 AC140484.1 SAMD14 IL1R2 C22orf29 TNPO3 HTT RAB3B CLINT1 CCDC53 FGF9 ZFC3H1 ARRDC3 STX12 SERPINB1 CASC5 HOMER1 PSAT1 CDH1 PLAG1 IFNG IQGAP1 CNTRL CREG1 CMPK1 SCN2A PUS7 GTF2IRD2 TSHZ3 DCTPP1 FIP1L1 SSH1 RHOB CAD GNPNAT1 DOCK10 KCNQ2 PDZD8 HTATIP2 PHYHIPL GNPTAB HADH INSL4 TRPM1 CACNG3 SASH3 NTN1 EPG5 HNRNPM IDI1 RRM2 ELOVL6 COG5 SACM1L EPHX1 BFAR ANKRD36BP1 KLHL8 UBE2E1 SLC30A10 ARAP2 AIMP1 DNAJC2 GADD45GIP1 LDB2 IGF1R SF3B1 ANKRD50 MTHFS CXCL9 IPO7 CAV1 STRBP BRSK2 HECTD3 HELLS TNS4 LHX6 ZNF584 STK4 ATPAF1 RAD54L JAM2 MICAL3 NCBP1 SNRPN FGFR1OP2 CACNA1S HDAC2 PRR7 ABCD1 TMCC1 LRRC40 CA2 TPO FAM32A CRYZ TTI2 MARC2 ASNS IL22RA1 CASZ1 HSP90B1 C1D HOXB6 USB1 PDHA2 SPC25 SGMS2 WDHD1 SLC30A6 TNFRSF10B SH2B3 TRIM44 KIF1C BTN3A3 TRAF1 COMMD3 RP11-89N17.1 SHFM1 SCAMP1 PKMYT1 TSEN34 DUT C1QB KIF18A MAP2 TNFRSF11A CYP51A1 IBTK TMEM170B PRLR MED28 STOML2 KCNA5 ALPI HSF1 TNRC6C POLR2H PKN1 ITM2A ARHGAP44 MLF2 MED9 SNORA70 SELT NSA2 TSC22D4 USP48 RRM1 C2CD2 FAM3A MFSD12 CTDSPL PDE11A BAZ1A MRPS2 CADM1 ZCCHC8 PRYP3 PTPRO ARID3B MEAF6 PPAT THUMPD1 KLHDC10 NIPA1 CALM3 IL7R PRSS16 PCCA SUGP2 RAB30 MPDZ OSBPL11 GNB5 ITFG1 IMPG1 C18orf25 SMN1 POU2F3 AKAP5 GBP3 NVL ATAT1 GGNBP2 ACVR1B CCDC15 ROM1 DSEL DTL ALG3 TMEM194A SRFBP1 MBIP COPB1 CREBRF RSAD2 RABL3 SMG6 INSR TGFBR2 PLEKHA8 TTN PDK1 WWTR1 DDX60L CIB1 CBY1 MDN1 OXSR1 REEP4 RAD51D RASAL2 ACN9 SLC38A1 KIAA2013 MMD NFKBIE PPFIA1 HBS1L TMEM30A WISP3 RNASET2 SOWAHC CHD5 MORC4 TCTN2 JAK1 ARFGEF1 TRAPPC4 CDK19 ZNF800 AUP1 GALNT11 LSM1 HIPK2 SLC25A28 CCND2 CAB39L CHERP ADIPOR2 AKT1 PEX11A PTPN3 NPTX1 CXCL6 SLC34A2 RNF125 ANKIB1 MCM6 NPTN EIF4A2 OR1A2 APOBEC3G MLNR MYPOP HSD17B12 UNC93B1 TMEM126B RSRC2 FDXR RAB27A FZD6 FBXO45 SLC25A12 ERCC6L PON2 YAP1 EDN2 PSMD7 ST8SIA2 RAD21 LRP4 HLA-DQB2 DYX1C1 ORC6 PNO1 ARL15 BCL3 SOX11 OR2F2 PTP4A1 ZMAT4 SMARCA2 ZNF791 TTPAL TRAPPC2P1 CST2 OAZ1 HIST2H2BF YIPF5 HSD11B1 S100A7A NAA25 KDM5D PSMB1 GMDS MIR22HG UBE2V2 EXOSC6 COL5A1 ZNF770 PAF1 DERL1 AGAP1 TMEM2 FKBP9 PMEPA1 TNFSF9 DCTD F3 TACO1 EIF4G2 CTSZ FAM208A RBMY1B KIF21B CDC5L ZNF250 PDSS2 ATE1 CLIC2 ARAF DCTN5 TEX30 KHDRBS1 DBR1 PEX5L ZNF324B UBR7 AP001324.1 NMD3 WNK1 C18orf54 ARL17A MED22 TSHB KIAA0754 MFHAS1 CSRNP2 NKX3-2 VPS13C KPNA6 HMGA1 ACOX2 GEM CA1 MFSD9 CYP4F11 ATG16L1 AGMAT JUN SERPINI2 NAGK DHODH B3GALT1 ADAMTS5 ATP2A2 ONECUT2 ZKSCAN7 CAST LGR4 TGFBI SULT1B1 UBE2D2 ATP1B3 NUP62 SCAF11 TMEM168 ZNF133 CISH GABRA3 GPR39 DHX36 PHTF2 APPL1 FLNA SNAP91 LRPAP1 TMF1 FAM160A1 NCOA4 RPS6KC1 CDH5 STXBP3 WHAMMP3 CYP24A1 FXYD3 SELENBP1 NDUFS7 MIB1 ALDH9A1 INPP5A COQ2 SRF SMARCE1 GHSR KHDC1 ZNF678 ICAM5 MAT2A TMEM106B MARCH1 TRAP1 WAPAL SMEK2 NPTXR SLC10A3 RNF6 BOLA2B VAV1 DPYSL3 ARMCX2 SEC22A DPY19L1 TOB1 TCEAL1 LMBR1 PRKAG1 IFI44 FAM49B LANCL1 FGF23 PCNT ELAVL3 EAF2 USP3 GNL2 ST18 ZNF200 FRMD4B FBLN5 ADRBK2 KRT32 KLHL24 PDE12 CNOT6L ARNT2 RPS6KB1 RAB8A TP53TG5 RXRB. | 2464 |
| **ebv-miR-BHRF1-2-5p** | SLC10A7 SLC25A46 PDCD4 GRB2 TMEM33 KDM4B PICALM UNG PALM2-AKAP2 AKIRIN1 PCBP1 MKLN1 GRPEL2 ZKSCAN1 BROX PRDM1 ZNF608 DEPDC1 GFPT1 F13A1 PUM2 DAZAP2 CREB1 BLCAP HMG20A ZNF544 DNAJC27 MACC1 STAU1 GAB3 ISOC1 CLEC2D CUTC TRPV3 PDCD1LG2 NOD2 BCL10 MYO1D ZNF850 MALT1 DGKH TEFM TWF1 ATP5B PAG1 SPOPL RHOA ATP2C1 NR3C1 PROSER1 RIF1 PD-L2 SUMO3 ITGAV TTC5 MYO5A PELI1 KDM3B RAB39B IKZF2 TYW3 MRPL3 SP100 MAPK6 SNX27 LY75 JMY CCNG1 C21orf91 PD-L1 BRWD3 CRK RNF149 SSX2IP CLIP1 TMEM254 ZNF12 ATIC. | 78 |
