# Supplementary material for: Lymphocyte to monocyte ratio predicts survival and is epigenetically linked to miR-222-3p and miR-26b-5p in diffuse large B cell lymphoma
Source: Sci Rep. 2023 Mar 25;13:4899. doi: 10.1038/s41598-023-31700-x (PMC10039925; doi:10.1038/s41598-023-31700-x)
Supplement: Supplementary file 10 — Supplementary Information 10. [file 41598_2023_31700_MOESM10_ESM.docx]

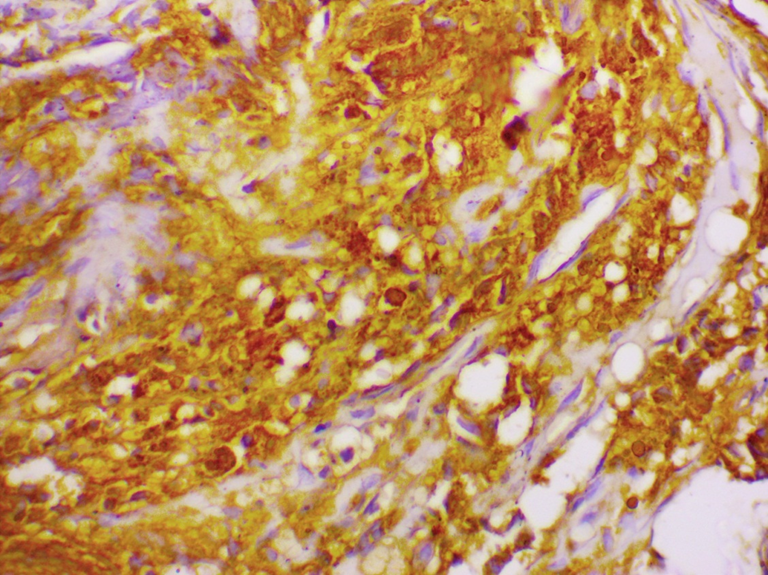


Supplementary figure S1a: IHC Positive reaction for BMI-1 immunostaining BMB section 400x.

Immuno Histo-Chemistry (IHC), B lymphoma Mo-MLV insertion region 1 homolog (BMI1), Bone Marrow Biopsy (BMB).


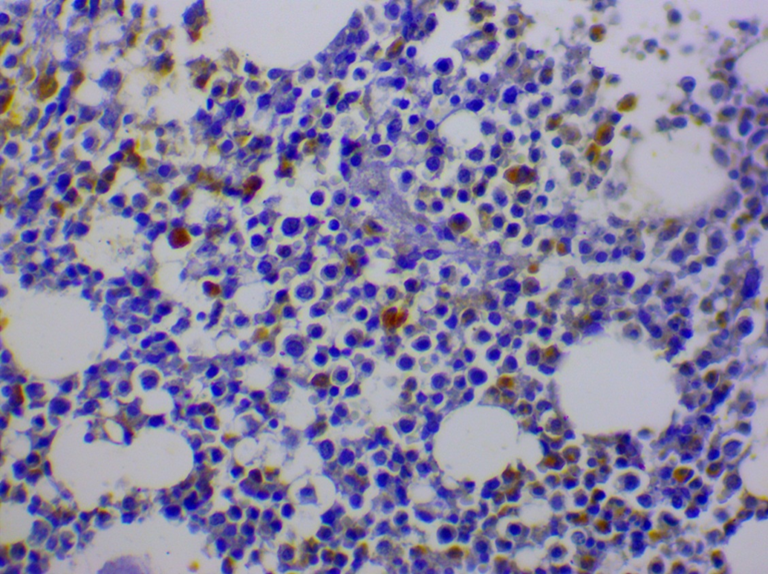


Supplementary figure S1b: IHC negative reaction for BMI-1 immunostaining BMB section 400x.

Immuno Histo-Chemistry (IHC), B lymphoma Mo-MLV insertion region 1 homolog (BMI1), Bone Marrow Biopsy (BMB).


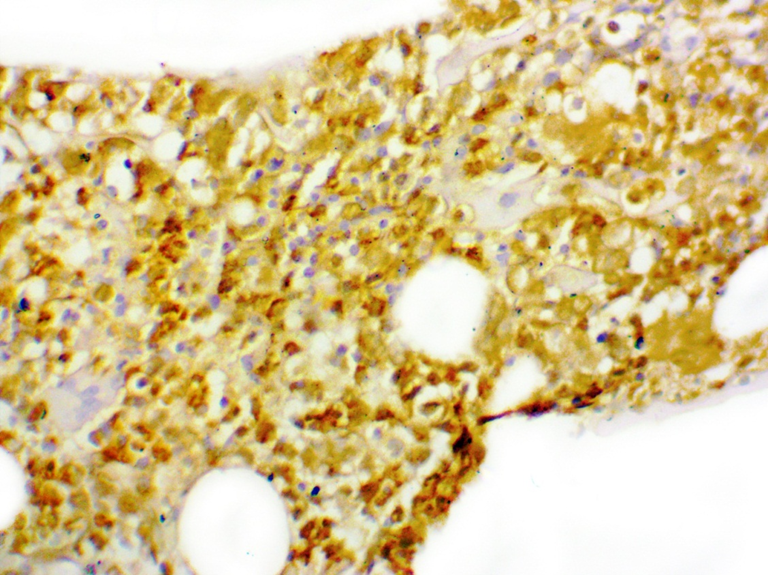


Supplementary figure S2a: IHC Positive reaction for PIM-2 immunostaining BMB Section 400x.

Immuno Histo-Chemistry (IHC), Prodigal insertion site in Maloney murine leukemia virus (PIM), Bone Marrow Biopsy (BMB).


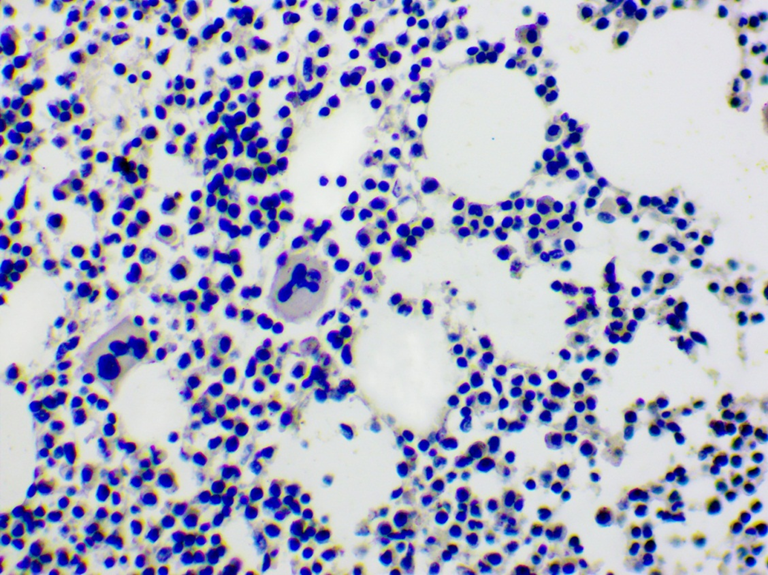


Supplementary figure S2b: IHC negative reaction for PIM-2 immunostaining BMB Section 400x.

Immuno Histo-Chemistry (IHC), Prodigal insertion site in Maloney murine leukemia virus (PIM), Bone Marrow Biopsy (BMB).
