# Supplementary figures and images for: Lymphocyte to monocyte ratio predicts survival and is epigenetically linked to miR-222-3p and miR-26b-5p in diffuse large B cell lymphoma
Source: Sci Rep. 2023 Mar 25;13:4899. doi: 10.1038/s41598-023-31700-x (PMC10039925; doi:10.1038/s41598-023-31700-x)

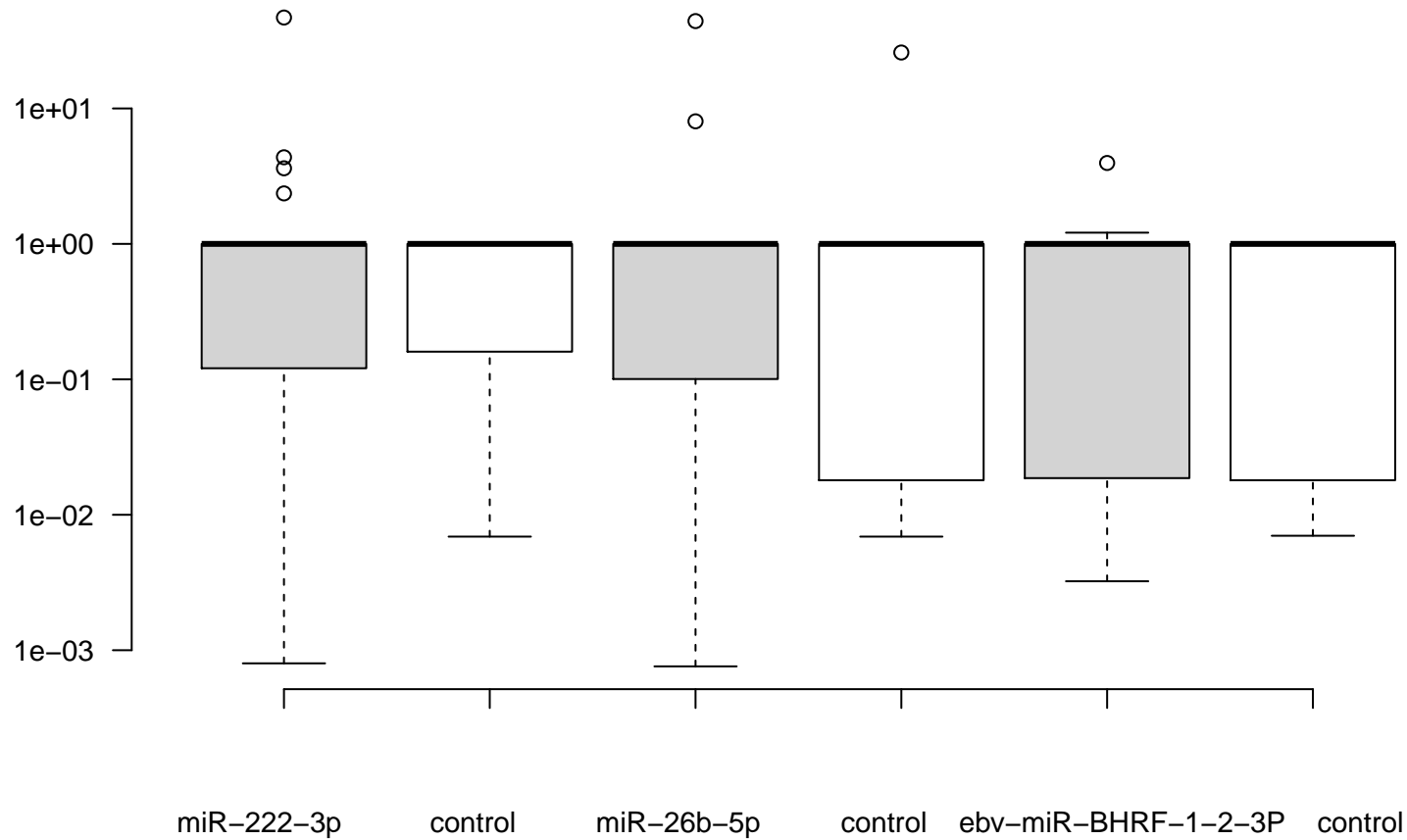

Fig.S3a: Expression levels of miR-222-3P, miR-26b-5p and ebv-miR-BHRF-1-2-3p in DLBCL and controls

Supplement: Supplementary file 11 — Supplementary Information 11. [file 41598_2023_31700_MOESM11_ESM.pdf]

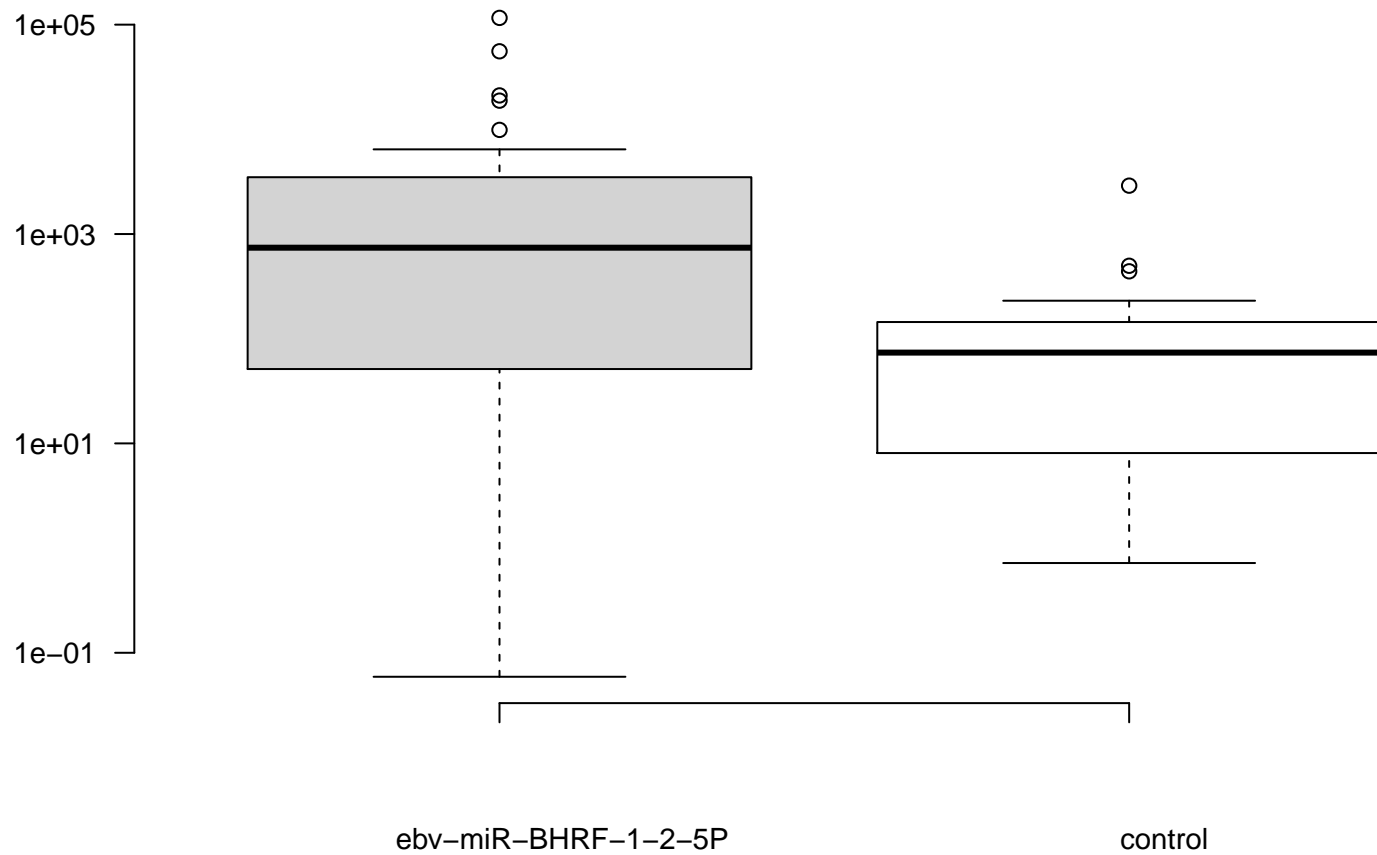

Fig.S3b: Expression levels of ebv-miR-BHRF-1-2-5p in DLBCL and controls.

Supplement: Supplementary file 12 — Supplementary Information 12. [file 41598_2023_31700_MOESM12_ESM.pdf]
