# Supplementary material for: Lymphocyte to monocyte ratio predicts survival and is epigenetically linked to miR-222-3p and miR-26b-5p in diffuse large B cell lymphoma
Source: Sci Rep. 2023 Mar 25;13:4899. doi: 10.1038/s41598-023-31700-x (PMC10039925; doi:10.1038/s41598-023-31700-x)
Supplement: Supplementary file 13 — Supplementary Information 13. [file 41598_2023_31700_MOESM13_ESM.docx]

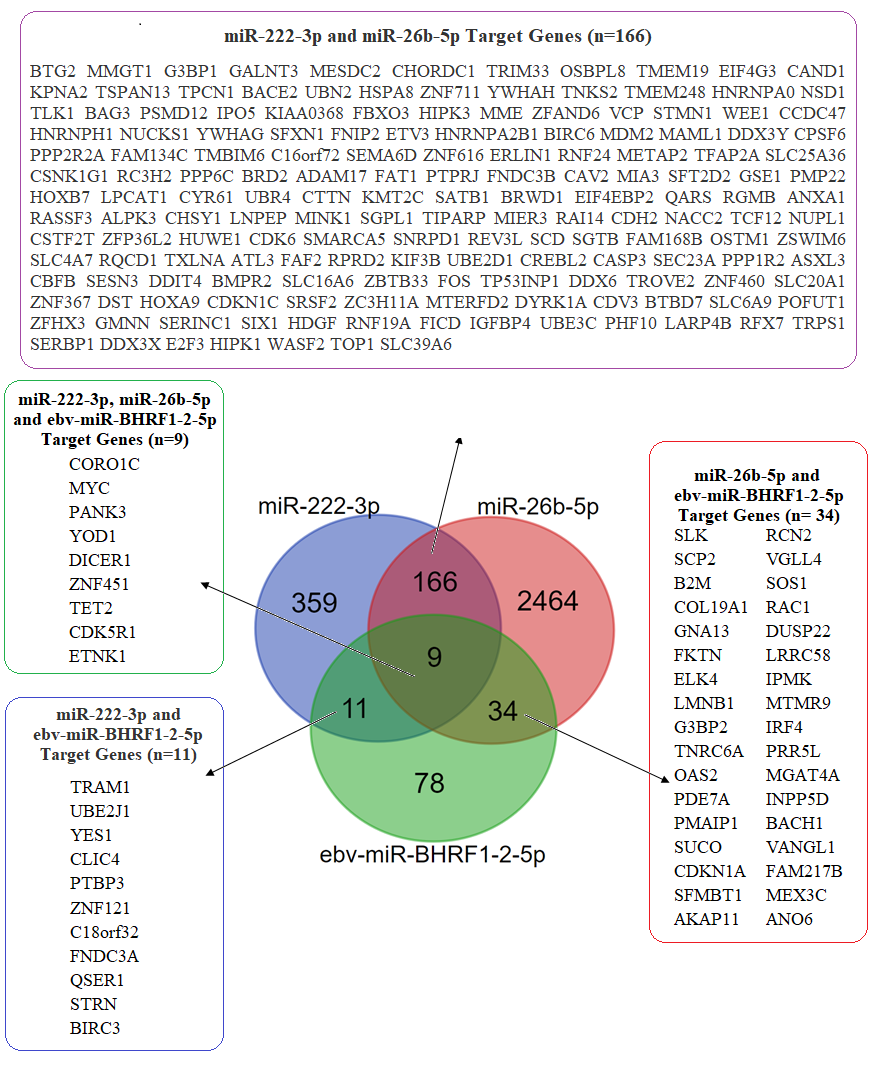


Supplementary fig. S4a. Venn diagram shows the overlapping and unique Tarbase experimentally supported interactions for hsa-miR-222-3p, hsa-miR-26b-5p and ebv-miR-BHRF1-2-5p.


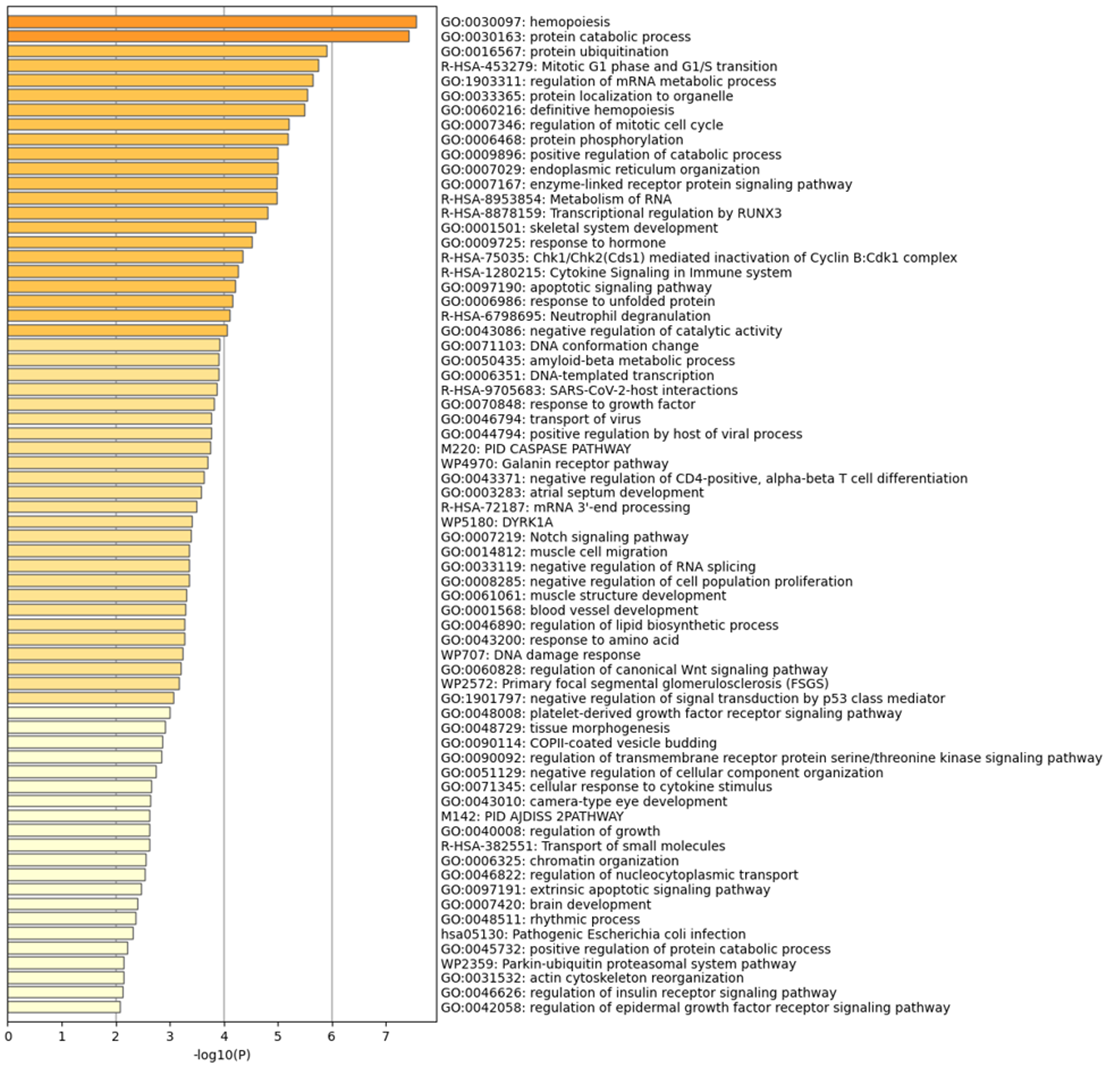


Supplementary fig. S4b: The enriched GO Biological Processes and Reactome Gene Sets for the overalpping 166 target genes of miR-222-3p and miR-26b-5p represented as bar-graphs, colored according to their p-values generated by Metascape.


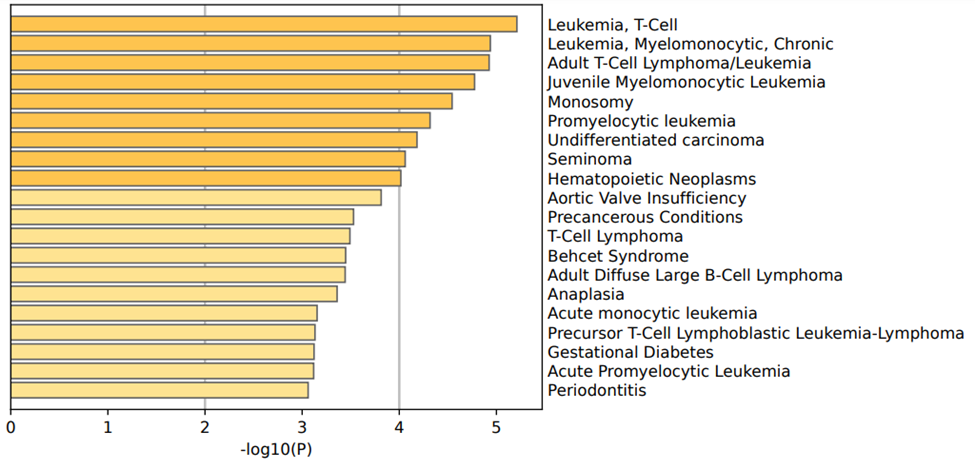


Supplementary fig. S4c. Enrichment analysis in DisGeNET for the 9 overlapping target genes of miR-222-3p, miR-26b-5p and ebv-miR-BHRF1-2-5p (CORO1C, MYC, PANK3, YOD1, DICER1, ZNF451, TET2, CDK5R1 and ETNK1). The top 20 human gene-disease associations (GDAs) represented as bar-graphs, colored according to their p-values.
